# Autophagy modulating agents as chemosensitizers for cisplatin therapy in cancer

**DOI:** 10.1007/s10637-020-01032-y

**Published:** 2020-11-07

**Authors:** Bartosz Mateusz Gąsiorkiewicz, Paulina Koczurkiewicz-Adamczyk, Kamil Piska, Elżbieta Pękala

**Affiliations:** Department of Pharmaceutical Biochemistry, Faculty of Pharmacy, Jagiellonian University Medical College, Medyczna 9, 30-688 Kraków, Poland

**Keywords:** Cisplatin, Autophagy, Cancer resistance, Combination therapy

## Abstract

Although cisplatin is one of the most common antineoplastic drug, its successful utilisation in cancer treatment is limited by the drug resistance. Multiple attempts have been made to find potential cisplatin chemosensitisers which would overcome cancer cells resistance thus improving antineoplastic efficacy. Autophagy modulation has become an important area of interest regarding the aforementioned topic. Autophagy is a highly conservative cellular self-digestive process implicated in response to multiple environmental stressors. The high basal level of autophagy is a common phenomenon in cisplatin-resistant cancer cells which is thought to grant survival benefit. However current evidence supports the role of autophagy in either promoting or limiting carcinogenesis depending on the context. This encourages the search of substances modulating the process to alleviate cisplatin resistance. Such a strategy encompasses not only simple autophagy inhibition but also harnessing the process to induce autophagy-dependent cell death. In this paper, we briefly describe the mechanism of cisplatin resistance with a special emphasis on autophagy and we give an extensive literature review of potential substances with cisplatin chemosensitising properties related to autophagy modulation.

## Introduction

### Cisplatin – General description

Cisplatin (cis-diamminedichloroplatinum(II)) is a square-planar geometry platinum coordination compound synthesized for the first time in 1844. Its cytotoxic properties were discovered in the 1970s and since its approval by FDA for cancer therapy in 1978 it has become one of the most commonly used drugs in the treatment of human neoplasm [1]. The classical view on cisplatin cytotoxic activity emphasizes its interaction with DNA. Cisplatin forms different types of adducts with DNA (monoadducts, intrastrand crosslinks, DNA-protein crosslinks) triggering DNA damage response, cell cycle arrest, and apoptosis [1]. Additionally, the role of mitochondrial damage resulting in excessive reactive oxygen species (ROS) generation and lipid peroxidation was highlighted [1]. Altogether these cellular events trigger intrinsic (mitochondrial) apoptotic pathway characterized with cytochrome c release and apoptosome formation leading to caspase activation. Cisplatin was also implicated in cell membrane fluidification which triggers non-specific Fas receptor activation and leads to extrinsic apoptotic pathway. Other mechanisms of cisplatin toxicity involve disruption of calcium signaling and Na+/H+ membrane pump and Na+/K+ ATPase inhibition [1–3]. Additionally due to its great reactivity cisplatin may bind to various proteins including enzymes thus modulating their activity [4].

Cisplatin is administered intravenously. Cellular uptake is mainly dependent on passive diffusion through the plasma membrane corresponding to 50% of drug transport [5]. Other possible routes include carrier-mediated transport (via CTR1 and OCT1–3), fluid-phase endocytosis as well as the internalisation of cisplatin-bounded transmembrane proteins [5]. In the cytosol, due to relatively lower chloride anion concentration, cisplatin becomes activated – one or two chloride ligands of platin are replaced by water ligands. This event not only potentiates cisplatin biological activity but also entraps molecule inside the cell. Activated cisplatin reacts with various substrates including protein sulfhydryl group and nucleic acids [1, 5]. Only a small portion of cisplatin (~1%) reaches the nucleus to bind DNA whereas the rest remains bounded with cytosolic proteins or entrapped in cellular compartments as Golgi, lysosomes and secretory vesicles [5, 6].

Cisplatin alone or in combination is utilized in chemotherapy regimens including first-line treatment of lung, head and neck, breast, testicular, ovarian, cervical, prostate, and bladder cancer in approximately 50% of all cancer-patients [1, 7]. However, its use may be limited due to numerous undesirable effects including drug resistance and adverse reactions such as nephrotoxicity, gastrointestinal disorders, allergic reactions, reduced immunity and hearing loss especially in younger patients. Other platinum-containing anti-cancer drugs such as carboplatin or oxaliplatin were also developed and successfully implemented in clinical use [1, 4]. The severity and duration of the aforementioned adverse effects depend on a lifetime cumulative dose of cisplatin which imposes a strict restriction on drug-based regimens. Additionally, tissue damage may become irreversible causing permanent disability [8]. Clinically implementation of combination therapy with cisplatin successfully reduces toxicity and resistance [1]. Up to this date, many substances were found to synergize with cisplatin anticancer activity as well as to reduce cancer cisplatin resistance in vitro and in vivo. Clinical studies indicate that the addition of 5-fluorouracil, paclitaxel, gemcitabine, doxorubicin to cisplatin results in a favourable outcome or reduced adverse effects in the treatment of various neoplasms [9]. However in this case it is difficult to conclude if the effect depends on drug synergism or resistance development restriction. Nevertheless available evidence encourages the search for new compounds with cisplatin chemosensitising properties that may improve cisplatin therapy efficacy.

### Mechanisms of cisplatin resistance in cancer cells

Antineoplastic drug resistance in cancer cells is a multidimensional phenomenon often difficult to tract. However, research in this area is required as it may uncover potential targets for novel cancer co-therapeutics acting synergistically with antineoplastic drugs as chemosensitisers.

Cisplatin resistance was widely explored in the past with many mechanisms found to play a role. The following sections will briefly summarize molecular alteration leading to resistant phenotype in cancer cells. This includes (i) alteration in DNA metabolism, (ii) epigenetic and transcription programs changes, (iii) increase in drug detoxification pathways activity, (iv) disturbed drug localization and trafficking (Table 1) [10].

**Table 1:** Cisplatin resistant phenotype in cancer cells may result from: (i) alteration in DNA metabolism, (ii) epigenetic and transcription programs changes, (iii) increase in drug detoxification pathways activity, (iv) disturbed drug localization and trafficking. Molecular mechanisms involved in each phenomenon and their effects on cells are summarised in the following table

Mechanism of resistance	Effect	Involved molecular mechanism
Alerted DNA metabolism [1, 10–12]	An increase in DNA repair machinery activity directly protects genomic DNA from cisplatin effects.	Increased activity of: nucleotide excision repair (NER), homologous recombination (HR), nonhomologous end joining (NHEJ), Fanconi anaemia pathway translesion synthesis (TLS). Mismatch repair (MMR) deficiency.
Changes is epigenetics and transcription profile [10]	Altered expression of cisplatin resistance phenotype proteins: involved in cisplatin trafficking (CTR1, TMEM205, ATP7A and ATP7B), transcription regulators (f.i. histone H1 and H3, SIRT1, GCF2, Nrf2, Snail, TWIST), small GTPases (Rab5, Rac1, RhoA, Rab8), cytoskeletal proteins, endocytosis/exocytosis regulators (ERC, STX6), chaperones (HSP 10, 27, 60 70, 90), ribosomal proteins and others.	Overexpression of chromatin remodeling enzymes as Tip60 acetyl-transferase or histone deacetylases 1, 3 and 4. The activity of histone demethylase as RBP2/KDM5A/Jarid1A - required for cisplatin-tolerance phenotype. Hypermethylation of promoter regions - reduced gene transcription and contributing drug resistance. Among others p53, p73 and insulin-like growth factor-binding protein-3 promoters’ hypermethylation were strongly correlated with cisplatin resistance.
Drug detoxification [1, 10, 13]	Cisplatin chemical deactivation and ROS scavenging.	Glutathione sulfhydryl groups are highly reactive toward cisplatin thus sequestering it and limiting its accessibility. Glutathione and proteins like thioredoxin and peroxiredoxin limit oxidative stress caused by cisplatin. Carbonyl reductase (CBR1), aldo-keto reductases - AKR1C1 and AKR1C3 activity was implied in resistance-phenotype development.
Drug trafficking and subcellular localization [1, 6, 10]	The decreased fraction of cisplatin able to interact with its molecular targets.	CTR1 downregulation limits cisplatin efflux and generates cisplatin-resistant phenotype. Increased expression of cell membrane (ATP7B and MRP 1–5) or vesicular (ATP5A) transporters augments active cytoplasmic efflux of cisplatin in resistant cells. Increased cisplatin accumulation in cellular compartments as Golgi, lysosomes, melanosomes and exosomes has recently gained attention as a potential mediator of cisplatin resistance.

**Table 2:** List of compounds reported to sensitize cancer cells towards CPT in the mechanism of autophagy/lysosomes modulation. A brief characterization of compounds, their cellular effects, and main findings from reviewed research are presented.

**L.p**	**Compound**	**Pharmacological/biological activity**	**Type of study**	**CPT chemosensitization - mechanism of action**	**References**
***Classical autophagy inhibitors***					
1.	Chloroquine and its derivatives (CQ)	- multi-active quinoline derivative - anti-inflammatory, anti-viral and anti-parasitic activity - autophagosome-lysosome fusion inhibitor - accumulates in lysosomes - triggers LMP	In vitro*:* NSCLC, oesophageal cancer, ovarian cancer, melanoma, urothelial carcinoma, gastric cancer, salivary gland carcinoma, endometrial cancer and tongue squamous carcinoma In vitro*:* glioblastoma, pediatric medulloblastoma cell lines and atypical teratoid/rhabdoid tumor In vivo: mouse xenograft CPT-resistant oesophageal, melanoma and hepatocarcinoma cancer model	- **↑** CPT induced apoptosis and/or senescence - loss mitochondrial membrane potential - **↑** caspases 8,3 level - selectivity in some experiments - no effect in normal cell lines - **↑** autophagy - **↓** late stage autophagy flux **- ↑**ROS - no influence - **↓** tumor mass	[36–57]
2.	Bafilomycin-A1(Baf-A1)	- a selective inhibitor of V-ATPase ATP6V0C/V0 subunit c - SERCA Ca2+ pump inhibitor - inhibits -autophagosome-lysosome fusion and/or lysosomal digestion	In vitro: bladder, oesophageal and cervical cancer, tongue squamous cell carcinoma	- **↓** of lysosomal CPT uptake thus **↑** the DNA-bound CPT portion - **↑** lysosomal biogenesis by c-Abl/TFEB pathway	[41, 51, 58–61]
3.	3-Methyladenine (3-MA)	- non-specific phosphosphatidylinositol 3-kinases (PI3K) inhibitor	In vitro: cervical cancer, gliomas, salivary adenoid cystic carcinoma, osteosarcoma, ovarian cancer, NSCLC, nasopharyngeal carcinoma, laryngeal cancer, hepatocarcinoma and urothelial carcinoma In vitro*:* tongue squamous cell carcinoma In vivo: Human Salivary Adenoid Cystic Carcinomaxenograft models	- **↑** caspase-dependent apoptosis - mitochondria hyperpolarisation **- ↑** ER-stress markers which were linked to caspase-4 and caspase-3 activation - no effect **- ↓** tumor mass	[38, 40, 42, 45, 54, 56, 62–71]
4.	Wortmanin (WT)	- non-specificphosphosphatidylinositol 3-kinases (PI3Ks) inhibitor	In vitro: cholangiocarcinoma, NSCLC In vivo*:* cholangiocarcinoma xenograft model	**- ↑** intrinsic apoptotic pathway - acts selectively towards rapidly proliferating cells **- ↓** tumor mass	[44, 63]
***Compounds well specified molecular targets***					
5.	mTOR/PI3K inhibitors: PKI402 AZD2014	- mTOR is a regulator of cellular metabolism linking nutrient status and growth factor (GF) signaling with autophagy induction	In vitro: hepatocarcinoma In vitro: glioblastoma, NSCLC, normal cells	- **↑** lysosomal biogenesis (mtROS/TFEB) - **↑** lysosomes number - induced mitochondrial depolarization, - **↑**mtROS generation - **↑**apoptosis - **↓** cell death	[36, 44, 72]
6.	MAPK inhibitors (UO126)	- MAPKs are kinases responsible for extra and intracellular signal transduction, amplification and coordination	In vitro*:* NSCLC In vitro*:* oesophageal cancer	- upregulation of thymidylate synthase and thymidine phosphorylase that grants CPT resistance - **↓** autophagy - **↑** apoptosis - **↑** senescence - **↓**cellular growth - **↓**apoptosis - **↓**senescence	[51, 73]
7.	MPT0L145	- selective FGF-R inhibitor	In vitro*:* bladder cancer	- **↓** viability of in CPT-treated CPT-resistant bladder cancer (mitochondrial dysfunction, ROS production, and DNA damage) - **↑** incomplete autophagic flux	[74]
8.	Nimotuzumab	- anti-EGF-R monoclonal antibody commonly utilized in cancer therapy	In vitro*:* oesophageal squamous cell carcinoma	- **↑** sensitivity towards CPT or paclitaxel-induced viability reduction - **↑** autophagy	[75]
9.	PIK3C3/Vps34 inhibitor: SAR405	- PIK3C3/Vps34 is a lipid kinase implicated in vesicular trafficking and autophagosome maturation	In vitro*:* urothelial carcinoma	- **↓** cell viability	[42, 76]
10.	Cepharanthine	- cholesterol trafficking inhibitor targeting Niemann-Pick disease type C1 (NPC1) protein - preventing cholesterol efflux into the cytoplasm - disruption of lysosomes	In vivo*:* breast and lung cancer xenograft models	- synergize with CPT to **↓** tumor growth	[77]
***Compounds influencing cellular metabolism***					
11.	Metformin	- antidiabetic drug - mitochondrial complex I and mitochondrial glycerophosphate dehydrogenase inhibitor - glutaminase inhibitor - **↑**β-oxidation, glucose uptake, glycolysis - antineoplastic activity	In vitro*:* breast and cervical cancer cells,	- **↓** in CPT induced autophagy **- ↑**apoptosis **- ↓** cancer cells proliferation - **↓** of autophagy secondary to **↓** of ammonia production	[78, 79]
12.	2-deoxy-D-glucose (2-DG)	- glycolysis inhibitor	In vitro: colon cancer, neuroblastoma, glioma grade IV	Cellular-specific effects: - **↑** endoplasmic reticulum (ER) stress and autophagy - **↑** apoptosis	[80, 81]
13.	Insulin	- a peptide hormone secreted by the pancreas implicated in glucose cellular uptake and metabolism regulation	In vitro*:* oesophageal squamous cell carcinoma	- **↑** Akt and mTOR expression and **↓** autophagy initiation which correlated with **↑ the** apoptotic ratio	[82]
14.	Nicotinamide Phosphoribosyltransferase inhibitor: FK866	- inhibits enzyme the catalyzing conversion of nicotinamide to nicotinamide mononucleotide	In vitro: neuroblastoma and cervical cancer	- **↑** cell death	[83]
***Natural compounds and their derivatives***					
15.	Oridonin	- bioactive ent-kaurane diterpenoid, a major active constituent of Rabdosia rubescens, which has been widely used in traditional Chinese medicine (anti-cancer, anti-inflammatory)	In vitro*:* *ovarian cancer, NSCLC*	- **↑**apoptosis - **↓** Belin-1 level - **↓** autophagy - limits adverse effects of therapy (CPT-induced nephrotoxicity in vivo in mice)	[83, 84]
16.	Phenoxofiol (PXD)	- an isoflavone analog with widely proved anticancer activity - inhibitor of apoptosis protein (XIAP) and FLICE inhibitory protein (FLIP) - direct inhibition of topoisomerase II and ENOX2 (ecto-NOX disulfide-thiol exchanger 2)	In vitro*:* ovarian cancer	- XIAP downregulation - **↓** autophagy - **↓** Beclin-1 level - limits adverse effects of therapy (CPT-induced neurotoxicity in vitro)	[47, 85, 86]
17.	Andrographolide	- naturally occurring labdane diterpenoid - anti-inflammatory, antiviral, antioxidant and anticancer action - may disrupt autophagosome-lysosome fusion - activator of Nrf2	In vitro*:* NSCLC and colon cancer In vivo*:* NSCLC xenograft model	- **↑** apoptosis - **↓** PTEN - **↑** lifespan	[87–89]
18.	4-Acetylantroquinonol	- tetrahydro ubiquinone derivative found in Antrodia camphorate, mushroom popular in Taiwan and Chinese medicine - anti-cancer activity	In vitro*:* ovarian cancer cell lines In vivo*:* ovarian cancer xenograft models	- **↑** apoptosis - **↓** autophagy - **↓** Akt and mTOR activity - **↓** tumor growth	[46]
19.	Pristimerin	- quinonemethide triterpenoid with anti-cancer activity isolated from Celastraceae and Hippocrateaceae - proteasome and telomerase inhibitor - **↓** MEK/ERK, EGF-R, PI3K/Akt, Wnt/β-catenin, NfxB - **↑** JNK - **↓** autophagy/**↑** autophagosome accumulation	In vitro*:* NSCLC In vivo*:* NSCLC mice xenograft model	- **↓** miR-23a - **↓** Akt and GSK3B phosphorylation - **↓** autophagy - **↓**tumor growth	[90–92]
20.	Icariin	- flavonoid usually derived from Epimedium sagittatum - anti-cancer, anti-inflammatory, anti-oxiant, anti-apoptotic	In vitro*:* multidrug-resistant ovarian cancer cell	- **↑** apoptosis - **↓** autophagic flux	[93]
21.	Melatonin	- human hormone regulating the sleep-wake cycle - antioxidant - Nrf2 activator - **↑/↓** autophagy (may be cell line specific)	In vitro*:* cervical cancer and head and neck squamous cell carcinoma	- **↑** apoptosis - **↓** mitophagy (secondary to **↓** JNK/parkinin activity) - **↑** mitochondrial ROS	[94–96]
22.	Procyanidins (OCP)	- Flavonoids - anti-oxidative, anti-inflammatory, antimicrobial, antiviral and anti-cancer activity	In vitro*:* laryngeal carcinoma	- **↑** apoptosis - **↑** autophagy	[97]
23.	Neferine	- bisbenzylisoquinoline alkaloid with anti-cancer activity derived from Lotus seeds - P-glycoprotein 1 inhibitor	In vitro*:* NSCLC	- **↑** ROS generation - **↓** PI3K/Akt/mTOR pathway - **↑** autophagy (**↑**LC3B-II/LC3B-I ratio) - **↓** Beclin-1 and PI3KCIII	[98, 99]
24.	Hyperoside	- flavonol glycoside present mainly in members of Hypericum and Crataegus genera - anti-inflammatory, anti-oxidant and anticancer activities	In vitro*:* ovarian cancer	- **↑** apoptosis - **↑** autophagy - selective towards CPT-resistant cells characterized with PGRMC1 expression and autophagic flux	[100]
25.	Bu-Zhong-Yi-Qi Decoction (BZYQD)	- Chinese herbal medication comprising extracts from six different herbs - anti-cancer activity	In vitro: NSCLC	- **↑** apoptosis - **↑** autophagy (accumulation of LC3-II and Atg7) - **↑** ROS generation	[101]
26.	Monanchocidin A (MonA)	- an alkaloid isolated from marine sponge *Monanchora pulchra* - cytotoxic properties in cancer cell lines	In vitro*:* resistant germ cell tumor cell line, prostate and bladder cancer	- selectivity (cancer specific) - **↑** cell death - **↑** unselective autophagic protein degradation - **↑** LMP at higher concentrations	[102]
27.	(−)-Epigallocatechin gallate (EGCG)	- polyphenolic catechin - anti-cancer activity in vitro	In vitro*:* colorectal cancer	- **↓** cancer cells proliferation - **↑** cell death - **↑** autophagosome formation and accumulation	[103]
28.	Chalcone-24 (Chal-24)	- a member of chalconoids - anti-microbial, anti-inflammatory and anti-neoplastic activity	In vitro*:* NSCLC	- **↑** apoptosis - **↑** JNK/Bcl-2/Beclin 1 dependent autophagy induction	[104]
29.	Resveratrol	- polyphenolic compound - anti-oxidant, anti-inflammatory, cytoprotective, anti-neoplastic effect - pleiotropic biological activity	In vitro*:* NSCLC	- **↑** autophagic flux - **↑** apoptosis (**↑**Bax expression and **↓**Bcl-2 and Akt phosphorylation)	[105–107]
30.	Gambogic Acid (GA)	- a xanthonoid compound derived from *Garcinia hanburyi* - anti-cancer activity proved in vitro and in vivo	In vitro*:* NSCLC	- **↑** cancer growth inhibition - **↑** autophagy - **↓** Akt/mTOR pathway	[108]
31.	GMI -an immunomodulatory protein derived from Ganoderma microsporum fungus	- antineoplastic activity was proved in vivo after oral administration - **↑** ER stress/calcium/Akt/mTOR pathway and triggers autophagic cell death attributed to unfused autophagosome accumulation	In vitro*:* NSCLC	- **↑** apoptosis - downregulation of ERCC1, XPF, and survivin	[109]
32.	Glaucocalyxin B (GLB)	- diterpenoid with anti-cancer activity extracted from Rabdosia japonica.	In vitro*:* gastric cancer	- DNA damage - **↑** ROS production - **↑** autophagy	[110]
33.	Poly-unsaturated fatty acids (PUFAs): Arachidonic acid (AA), eicosapentaenoic acid (EPA), docosahexaenoic acid (DHA)	- multidirectional positive effects in human organism	In vitro*:* NSCLC, cervical cancer	- **↑** apoptosis (**↑**caspase 3/7 activity) - **↑** autophagy - **↓** cancer cells viability - **↓** caspase 3 and PARP cleavage, but with Bcl-2 downregulation and**↓** LC3B-II expression - limiting CPT-mediated nephrotoxicity in mice	[111, 112]
***Other Compounds***					
34.	Quinacrine (QC)	- anti-malarial drug - structurally related to CQ	In vitro*:* ovarian cancer and head and neck squamous cell carcinoma	- **↑** autophagic flux - **↑** autophagosome accumulation	[113, 114]
35.	Graphene oxide	- medical nanotechnology, (drug delivery systems) - may trigger LMO - subsequent **↑** autophagy induction and **↓** late-stage flux (**↓** lysosomal degradation)	In vitro*:* cervical, prostate, ovarian and colon cancer In vitro*:* NSCLC	- **↑** cell death - **↑** necrosis - **↑** autophagy - no influence	[115, 116]
36.	Proteasome inhibitor: bortezomib	- proteasomes are responsible for protein degradation - **↓** autophagic flux (**↓** cathepsin activity secondary to sustained ERK activation)	In vitro*:* ovarian cancer	- **↑** cell death	[117]
37.	Zoleandronic acid (ZA)	- treatment of multiple bone disorders as osteoporosis and bone metastasis	In vitro*:* salivary adenoid cystic carcinoma cell line	- **↑** apoptosis - **↑** ROS production - LC3B autophagy marker accumulation	[118]
38.	Lithium (Li)	- multiple biological effects - **↑** autophagy induction **-** late-stage autophagy inhibition and vesicles accumulation	In vitro*:* oesophageal and colorectal cancer	- **↑** cell death - accumulation of cytoplasmic vesicles - LMP induction	[119]
39.	C60(Nd) nanoparticles (C60(Nd))	- medical nanotechnology, (drug delivery systems)	In vitro*:* cervical cancer	- **↑** cell death	[120]

#### Alerted DNA metabolism

An increase in DNA repair machinery activity may directly protect genomic DNA from cisplatin effects. Among other pathways, nucleotide excision repair (NER) seems to play a pivotal role. NER facilitates cisplatin adducts excision and DNA repair and its activity positively correlates with cisplatin resistance [1, 10, 11].

Interestingly another mechanism responsible for single-strand DNA damage repair - mismatch repair (MMR) plays an opposite role in cisplatin resistance. MMR machinery recognizes cisplatin lesion sites but is unable to repair hence shielding cisplatin adducts from NER mediated repair and promoting apoptosis. Consistently MMR deficiency promotes cisplatin resistance [11].

Besides NER other mechanisms as homologous recombination (HR), nonhomologous end joining (NHEJ),Fanconi anaemia pathway and translesion synthesis (TLS) are implicated in alleviating cisplatin mediated genomic DNA damage thus contributing resistance [10–12].

#### Epigenetics and transcription profile alteration

Cisplatin resistant cancer cells are characterized by significant changes in transcription profile. This depends on epigenetic changes as an alteration in histone remodeling or DNA methylation [10].

Overexpression of chromatin remodeling enzymes as Tip60 acetyl-transferase or histone deacetylases 1, 3 and 4 were linked with cisplatin resistance [10]. Additionally, the activity of histone demethylase as RBP2/KDM5A/Jarid1A was found to be required for the cisplatin-tolerance phenotype [10].

Furthermore, cisplatin resistance development is linked with significant changes in DNA methylation landscape. Prolonged cisplatin treatment was shown to induce hypermethylation of many promoter regions leading to reduced gene transcription and contributing drug resistance. Among others p53, p73 and insulin-like growth factor-binding protein-3 promoters’ hypermethylation was strongly correlated with cisplatin resistance [10].

The aforementioned epigenetic changes lead to alteration in the transcription profile. Cisplatin resistance phenotype was linked with alerted expression of: proteins involved in cisplatin trafficking (CTR1, TMEM205, ATP7A and ATP7B), transcription regulators (f.i. histone H1 and H3, SIRT1, GCF2, Nrf2, Snail, TWIST), small GTPases (Rab5, Rac1, RhoA, Rab8), cytoskeletal proteins, endocytosis/exocytosis regulators (ERC, STX6), chaperones (HSP 10, 27, 60 70, 90), ribosomal proteins and others. Additionally emerging line of evidence indicates the importance of miRNA expression profile (miRNome) changes in cisplatin resistance [10].

#### Drug detoxification

Apart from cisplatin active efflux (discussed in the following section), intracellular detoxification depends on endogenous compounds know to inactivate cisplatin. Among those glutathione and metallothioneins seem to play a crucial role. Glutathione sulfhydryl groups are highly reactive toward cisplatin thus sequestering it and limiting its accessibility. Moreover, increase in both glutathione and proteins like thioredoxin and peroxiredoxin limits oxidative stress caused by cisplatin [1, 10]. The latter is greatly dependent on Nrf2 transcription factor induction [10]. Additionally, carbonyl reductase (CBR1), aldo-keto reductases - AKR1C1 and AKR1C3 activity was implied in resistance-phenotype development [13].

#### Drug trafficking and subcellular localization

Increased efflux and decreased influx are important and universal mechanisms of drug resistance. Although cisplatin uptake is mainly facilitated by passive diffusion, the role of protein interaction-mediated and active transport was also emphasized (see above). Consequently, CTR1 downregulation may limit cisplatin efflux and generate cisplatin-resistant phenotype [1, 10]. On the other hand, increased expression of cell membrane (ATP7B and MRP 1–5) or vesicular (ATP5A) transporters augment active cytoplasmic efflux of cisplatin in resistant cells [1, 10].

Moreover increased cisplatin accumulation in cellular compartments as Golgi, lysosomes, melanosomes and exosomes have recently gained attention as a potential mediator of cisplatin resistance [6].

#### The role of a vesicular compartment in antineoplastic drug resistance

The role of autophagy in antineoplastic drug resistance is vastly researched. Cisplatin treatment strongly induces cellular autophagy [14]. Although it was mainly considered as a protective mechanism linked to cisplatin-resistant phenotype recent line of evidence suggests that the autophagy role is context-dependent. In some conditions, cisplatin triggered autophagy may conversely enhance cell death [14]. Furthermore, the latter effect was shown to be enhanced by various chemical compounds. In result not only autophagy inhibitors but more generally its modulators are potential cisplatin chemosensitisers.

Moreover, cisplatin-resistant cancer cells are characterized by alterations in vesicular compartment functioning. These include the reduction of the lysosomal compartment with downregulation of LAMP-1 and 2 [15, 16]. Aberrant function and expression of the lysosomal H + -pump in cisplatin-resistant cells result in decreased lysosomal acidification [17]. This not only results in deficient lysosomal enzymes processing but additionally alerts lysosomal localization, trafficking, fusion and promotes lysosomal drug accumulation and exocytosis [15–18] The latter process may serve as an additional rout of drug efflux as cisplatin may be actively transported into lysosomes by ATP5A [5, 6]. Deficiency in lysosomal enzymes activity was also linked to decreased EGF degradation [17]. Lately, the role of Rab7A downregulation in cisplatin resistance was evaluated. Rab7A is a RAB GTPAse family member localized to late endosomes and lysosomes. Rab7A facilitates the maturation of early and late endosome, promotes lysosomal biogenesis, acidification, clustering and their fusion with late endosomes. Reduced expression of Rab7A was found in cisplatin-resistant cancer cells to directly confer drug resistance. Downregulation of Rab7A was linked with increased production of cisplatin loaded extracellular vesicles (EV) serving as an additional drug efflux route. Additionally, another study proved that cisplatin treatment induces EV release which serves as mediators of communication with tumor environment to induce invasiveness and drug resistance [19]. Of note, other studies found reduced expression of RAB5A and upregulation of RAB8A in cisplatin-resistant cells [16].

Furthermore, cisplatin resistance phenotype was linked with aberrations in the endocytic compartment. This includes disruption in endocytic recycling leading to in membrane proteins mislocalization. Moreover, cisplatin-resistant cells lose normal perinuclear localization of the endocytic recycling compartment (ERC) which becomes dispersed equally. This was suggested to depend on alterations in microtubules and to play a direct role in cisplatin resistance [20].

At last reduced fluid-phase endocytosis was suggested to reduce drug uptake in cisplatin-resistant cells [5].

### Autophagy and lysosomes

#### Autophagy in cancer

The role of autophagy in cancer is ambiguous and context-dependent. Autophagy may limit carcinogenesis through the elimination of damaged mitochondria (mitophagy) generating excessive amounts of ROS [14]. On the other hand, autophagy serves as a cellular mechanism to overcome environmental stress thus acting as a protective mechanism to promote tumor growth [14]. Specifically, autophagy induction was linked to the chemoresistant phenotype, cancer stemness, and dormancy [14, 21].

#### The biological role, molecular mechanism and regulation

**Fig. 1:**
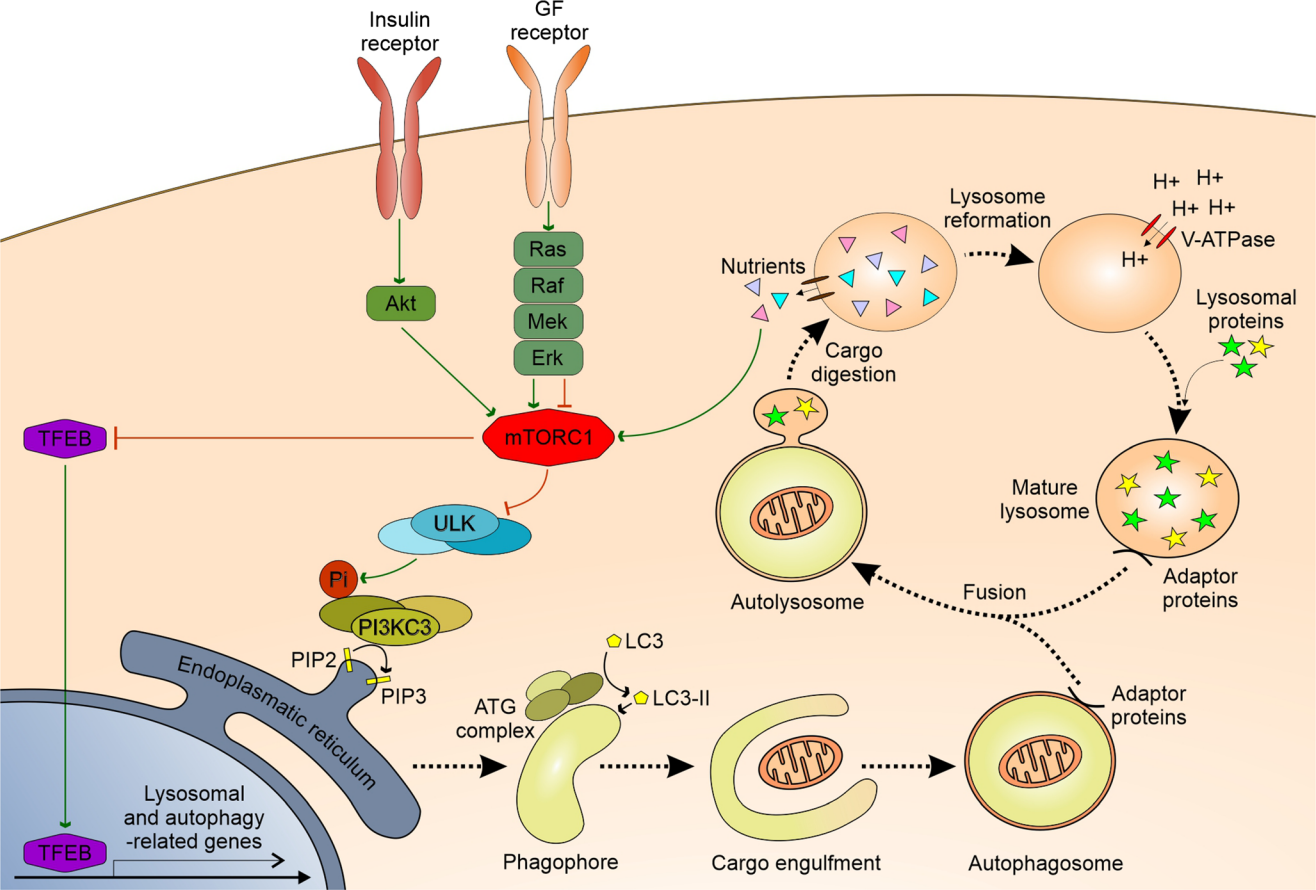
A simplified scheme depicting: autophagosome formation, autophagy process and lysosome reformation with associated regulatory pathways. A more detailed description is provided in the corresponding sections. Note that for convenience “ULK”, “PI3KC3”, “ATG” stands for entire complexes consisting of respective proteins. Utilized abbreviations stand for: GF – growth factor, Pi – phosphate group, PIP2 – phosphatidylinositol-(3,4,5)-triphosphate, PIP2 – phosphatidylinositol-(4,5)-bisphosphate. Green arrows symbolize activation processes whereas red blunt-ended lines correspond to inhibition. Other arrows depict the sequence of the process or transformation

Autophagy is a physiological and evolutionary conserved process leading to a controlled degradation of cell’s components. Although basic level of autophagy is maintained to remove damaged cellular components, it is significantly upregulated in response to various stressors. Autophagy is subdivided in three different types – macroautophagy, micorautophagy and chaperone-mediated autophagy [22]. In this review term autophagy will mostly concern macroautophagy unless otherwise specified. The latter two types are utilized autonomously by lysosomes either by direct engulfment of cytoplasmic cargo in the case of microautophagy or by translocation of soluble cytosolic proteins through specialized transporters in lysosome membrane [22]. The following sections are aimed to review the mechanism and regulation of autophagy. Additionally, a simplified scheme of the process is presented in Fig. 1**.**

Macroautophagy begins with the formation of prime vesicles – omegasomes on the membrane of endoplasmic reticulum (ER). This specific location at ER is primed by the presence of ATG9 protein. The active ULK1/2 (unc-51 like autophagy activating kinase) complex is recruited to omegasome causing phosphorylation of PI3K class 3 complex and following PI3P formation. Subsequent series of events leads to the recruitment of various autophagosome membrane protein (e.g. ATG5-ATG12/ATG16L and LC3 with its further conversion to LC3-II), the formation of phagophore vesicle, its elongation and maturation [23]. Phagophore engulfs cytoplasmic material either nonspecifically (bulk autophagy) or specifically. The latter is reserved for distinct structures as damaged mitochondria (mitophagy), protein aggregates and others. This depends on the interaction of LC3 autophagosome residual protein with “eat-me” signals (for instance ubiquitin chains, proteins with LC3-interacting region, or distinct lipids) presented on target structures [22, 24]. The next phagosome undergoes enclosure around engulfed cargo with the formation of the double-membrane autophagosome. To successfully retrieve materials from engulfed structures mature autophagosome need to fuse with the lysosome. This event delivers enzymes to digest the autophagosome inner membrane and its content. Successful fusion depends on tethering factors present on lysosomal and autophagosome membrane - HOPS complex, Rab7 and a set of adaptor proteins. Following tethering outer autophagosome membrane undergoes SNARE dependent fusion with lysosomal membrane forming autolysosome [23, 24]. After the breakdown of cargo by lysosomal hydrolases, nutrients are retrieved from the lumen of autolysosome by membrane transporters. At last lysosome reformation begins – vesicles rich in lysosomal membrane proteins buds from autolysosome outer membrane in a clathrin-mediated process called autophagic lysosome reformation (ALR) [23].

Proper maturation and microtubule transport of autophagosome are essential for autophagy realization and disruption of any may lead to stalling of the process. Similarly appropriate lysosomal trafficking is a prerequisite for fusion with autophagosome [23, 24]. In this context, it is worth mentioning that lysosome positioning alteration is one of cancer hallmarks [25].

Proper lysosomal activity is required for autophagy to succeed. Lysosomes are single membrane vesicular organelles containing acid hydrolytic enzymes implied in digestion - both extracellularly (after exocytosis) and intracellularly upon fusion with endosomes and autophagosomes. For the optimal activity of enzymes, lysosomal lumen pH is maintained at a low level by the presence of membrane V-ATPase. In addition to a conventional role in digestion, lysosomes play a vital role in metabolism regulation, cellular signaling, secretion and cell membrane repair [26].

Under specific circumstances, lysosome may fuse with cell membrane in an exocytosis process. This both supplies material for membrane repair and mediates secretion of lysosomal lumen content. The latter delivers proteolytic enzymes to cleave extracellular matrix which may promote cancer migration and progression. Additionally, lysosome exocytosis may serve as a paracrine mechanism through ATP transport [26]. Importantly this process is also related to multi-drug resistance in canner by contribution to drug efflux. Cytotoxic drugs, especially cationic amphiphilic compounds, are known to be sequestered in the lysosomal lumen and to trigger their exocytosis [27].

As mentioned above autophagy may be greatly enhanced in response to many cues which require strict regulation. This includes two master regulators – mTORC1 and TFEB. mTOR is a protein complex with kinase activity implied mainly in cellular metabolism and growth regulation. Active mTORC1 both phosphorylates and sequester ATG13 and ULK1 to diminish their activity, while mTORC1 inhibition (f.i. during starvation) promotes ATG13 and ULK1 activity, initiating autophagy [24]. Lysosomes serves as a scaffold for mTORC1. mTORC1 is recruited to the lysosome by lysosome membrane resident Rag GTPases upon their activation by nutrient flux. Additionally, another lysosomal GTPase – Rheb may allosterically activate mTORC1 in response to growth factor (GF) signaling. Importantly lysosomal tethering enables close coordination of nutrient sensing and growth factor signaling in mTORC1 activation [28]. On the other hand, TFEB is a transcription factor pivotal for the expression of both autophagy and lysosome biogenesis related genes [24]. Its activity is inhibited by mTORC1 and promoted by nutrient-deficiency mediated lysosomal calcium efflux. Off note TFEB signaling is also thought to be pivotal for lysosome exocytosis [26, 28].

Many feedback loops are involved in a proper autophagy regulation. For instance, mTORC1 deactivates TFEB. In turn, TFEB controls mTORC1 lysosomal recruitment and upregulates mTORC1 activating Ras-related GTP-binding protein D [24]. Other proteins implicated in autophagy regulation include AMPK, BCL-2, AKT and MAPK pathway components [22, 24].

#### Autophagy and lysosomes in cell death

For a long time, autophagy was considered as an exclusively cytoprotective mechanism and was implied to oppose apoptosis. Indeed autophagy and apoptosis are to some extent mutually exclusive. Autophagy limits apoptotic cell death by the elimination of mitochondria which prevents ROS generation and cytochrome-c release [29]. Conversely during the course of the apoptotic execution phase caspases cleave autophagy-related proteins thus inhibition the latter process [29]. Interestingly products of this cleavage often act proapoptotically in a positive feedback loop [29]. Additionally, sequestration of autophagy regulator protein - beclin-1 by antiapoptotic bcl-2, bcl-xl and Bim is vital for discussed crosstalk [29]. In opposition to its protective role autophagy may also contribute to cell death. Selective autophagy of apoptosis inhibitors as FoxO3a (transcription factor for proapoptotic Puma), Bruce (intrinsic apoptotic pathway inhibitor), Fap-1 (Fas-mediated apoptosis inhibition), or caveolin-1 promotes this modality of cell death [29–31]. Furthermore, selective autophagy of catalase promotes necrotic-like cell death and autophagy of ferritin causes ferroptosis – a type of cell death mediated by excessive free iron accumulation [31]. Interestingly autophagosome may serve as a scaffold for apoptotic or necroptotic machinery facilitating respectively FADD-caspase 8 and FADD-RIP1-RIP3 (necrosome) activation [31]. Additionally, mitophagy as well as stalled autophagy resulting from autolysosome formation failure may lead to cellular demise [32]. Importantly autophagy-dependent cell death may occur independently on other modalities of apoptosis, necroptosis or ferroptosis. This may be the result of excessive “bulk” autophagy or may depend on autosis - a distinct form of PCD involving the Na+/K+ ATPase [32].

At last, lysosomes are also implicated in programmed cell death. Current nomenclature guidelines define lysosomal cell death as dependent on primary lysosomal membrane permeability (LMP) and lysosomal acid proteases - cathepsin activity. LMP is characterized by comprised lysosomal membrane integrity which enables lysosome content leakage into the cytoplasm [33]. This is promoted by various factors including caspase activity, Bax, p53, ROS, membrane lipid changes, lysosomotropic agents (including some chemotherapeutic) and prevented by antioxidants, Bcl-2, Hsp70, LAMP-1/2 and membrane cholesterol [34]. As mentioned above cathepsins are the main executors of lysosome dependent cell death. Upon cytoplasmic translocation, they cleave various target proteins. Cathepsin activity results in degradation of antiapoptotic XIAP, Bcl-2, Bcl-xl and Mcl1 as well as inactivation of proapoptotic caspase8, Bid and Bak [34, 35]. The additional mechanism depends on calpain cleavage as well as on iron release and consequent ROS generation [34]. Whereas moderate LMP leads to apoptosis, a massive one may trigger necroptosis [33]. Lysosomes are an attractive target for antineoplastic therapy due to the convergence of many cellular processes and pathways dysregulated in cancer on those organelles.

## Literature review

### Rationale and methodology of the study

Cisplatin is currently one of the most important antineoplastic drugs in use. However high prevalence of primary and acquired cisplatin resistance is a vital limitation for cisplatin treatment regimes. The development of effective co-therapeutics capable of overcoming this phenomenon is a possible solution for improving clinical outcomes. This requires the knowledge of molecular mechanisms standing behind resistant phenotype and identification of potential targets. Simultaneously numerous reports indicate the importance of autophagy and lysosomes in cancer development and more specifically in drug resistance. These, combined with high autophagy inducing potential of cisplatin, prompted us to write this paper in a form of review on chemical compounds modulating autophagy and lysosomal physiology that sensitizes cancer cells to cisplatin.

We searched PubMed database for entries containing experimental data linking modulation of autophagy/lysosomes with chemical compounds (excluding pure gene manipulations) and cisplatin treatment efficacy in vitro or in vivo. We identified 39 chemical compounds capable of cisplatin chemosensitization (Table 2). In the following sections, we will briefly main findings from the research.

### Compounds

#### Chloroquine and its derivatives

Chloroquine (CQ) is a multi-active quinoline derivative with anti-inflammatory, anti-viral and anti-parasitic activity [36]. CQ is a weak base capable of passive diffusion through cell membrane. It accumulates in acid intercellular vesicles like lysosomes where it becomes protonated and entrapped. Experimentally CQ is commonly utilized as a late-stage autophagy inhibitor. CQ inhibits lysosome–autophagosome fusion (while not significantly affecting lumen pH value) which may be due to disruption of SNAP29 recruitment to lysosomal membrane [36, 37]. This may cause autophagic flux stalling with autophagosomes accumulation leading to autophagy-dependent cell death. Moreover, LC-3 lipidation promotion by CQ induced osmotic imbalance may affect autophagic flux [37]. Additionally CQ treatment leads to lysosomal clustering and enlargement as well as to disorganization of Golgi complex and endo-lysosomal system expressed in disruption of EGF-R endocytic transfer [37]. Moreover, after intralysosomal accumulation CQ may destabilize the lysosomal membrane leading to LMP (preferentially in cisplatin-resistant cells characterized with increased lysosomes number) due to its detergent-like properties. The latter is most visible during treatment with high concentrations of CQ (40–160 μM) whereas at lower concentrations (10–20 μM) autophagy inhibition is a predominant effect [48].

CQ or HCQ were shown to potentiate CPT anti-neoplastic effect in multiple cancer cell lines [38–49, 51–57].

CQ enhances CPT induced apoptosis and/or senescence. CQ treatment was also linked to loss of mitochondrial potential as well as to caspase 7 and 3 activation implicating the role of intrinsic mitochondrial apoptotic pathway [45, 48, 54, 55].

CQ chemosensitizing properties manifest with selectivity in some experiments. CQ effect on CPT cytotoxicity was lesser in normal and CPT-sensitive cancer cell lines [43, 48, 54]. However other experiments do not support this conclusion [38, 41, 42, 53]. Additionally, no influence on CPT treated cell viability was also observed for several types of cancers (glioblastoma, pediatric medulloblastoma cell lines and atypical teratoid/rhabdoid tumour cell lines) [50]. Circu et al. found that a low dose of CPT (25 uM) is inefficient in enhancing CPT activity in resistant A549 cell line though it successfully inhibits autophagy. On the other hand, high concentrations (100 uM) of CQ synergized with CPT in cell viability reduction. The latter effect was suggested rather to depend on LMP induction than autophagy inhibition. Conversely in the same experiment ATG5 knockout efficiently reversed CPT resistance. It was suggested that CQ at low concentrations may exhibit additional side mechanism limiting autophagy-inhibition induced cancer cells chemosensitization [48]. On the other synergistic effect of CQ and CPT was proved in other studies for CQ at concentrations as low as 1uM in ovarian cancer and at 4 μg/mL (~12,5 uM) in a resistant A549 cell line [43, 53]. Additionally, xenografts experiments proved that CQ administrated peritumourally is in enhancing CPT mediated tumor mass reduction in CPT-resistant oesophageal cancer, melanoma and hepatocarcinoma [45, 51, 52]. In the first case, CPT treatment alone was totally inefficient.

Clinically co-administration of CPT and member of tyrosine kinase inhibitors (TKI) such as gefitinib or erlotinib was shown not to improve outcomes. This may result from TKI mediated induction of autophagy which generates CPT-resistant phenotype. CQ addition to gefitinib and CPT regiment was shown to overcome this antagonistic effect enhancing cell death in NSCLC cell line [39].

CPT resistance correlates with high basal autophagy. Consistently ATG genes knock down and CQ treatment sensitizes cancer cells to CPT [48, 51, 53]. Subsequently ATG5/7 knockout inhibits CQ (in monotherapy) antineoplastic activity [43]. This suggests that although autophagy induction itself acts as a CPT-protective mechanism, it is necessary for CQ activity. As mentioned above in some cases CPT resistant cell lines characterized with the higher basal level of autophagy were more prone for CQ mediated growth inhibition. Simultaneous autophagy induction by CPT and late stage autophagy flux inhibition with consequent accumulation of unfused autophagosomes and lysosomes may play a vital role in CQ-CPT synergism.

Off note experiments with oxaliplatin (OXA) proved that CQ or 3-MA addition augments OXA induced ROS generation. ROS scavenging with N-acetylcysteine, significantly reduced OXA-CQ/CPT-3-MA induced cell death. Given the similarity of OXA and CPT ROS may play an important role in CQ mediated chemosensitization [57].

Hydrochloroquine (HCQ) is a less toxic derivative of CQ with similar clinical applications. HCQ cellular effects are similar to those of CQ and consistently its was shown to possess CPT chemosensitizing properties in vitro [46, 47, 49].

#### Bafilomycin-A1

Bafilomycin-A1 (Baf-A1) is a macrolide considered to be a selective inhibitor of V-ATPase ATP6V0C/V0 subunit c. Its action results in decreased proton flux into lysosomal lumen thus decreasing their acidification. This disrupts proper lysosome functioning (specifically lysosomal enzymes activation) and autophagic flux [58]. Bafilomycin-A1 inhibits lysosome-autophagosome which is secondary to lysosomal deacidification or Ca2+ pump SERCA inhibition [59]. Additionally, there is evidence for BafA1 mediated disruption of endocytic compartment [58].

In vitro Baf-A1 successfully mediated CPT-chemosensitisation of: bladder, oesophageal and cervical cancer as well as tongue squamous cell carcinoma cell lines [41, 51, 60, 61]. In the latter case, Baf-A1 and CPT synergistic effect was shown to rely on the inhibition of lysosomal CPT uptake thus enhancing the DNA-bound CPT portion. Importantly autophagy inhibition by ATG5 knockout or 3-MA did not replicate this synergism suggesting that autophagy is not the primary target of Baf-A1. Consistently co-treatment with CPT and Baf-A1 did not significantly influence autophagic flux measured by LC3-II protein level. Conversely, CPT induces lysosomal biogenesis by c-Abl/TFEB pathway and TFEB knock-down successfully increased the cytotoxic effect of CPT [60]. Moreover, Leisching et al. proved Baf-A1 addition to nontoxic CPT concentrations significantly enhanced cell death in cervical cancer cell lines while subsequently protecting normal cervical epithelial cells from CPT cytotoxic effect. This was correlated with a higher level of basal autophagy in cancerous cell lines [61].

#### 3-MA

3-Methyladenine (3-MA) is a non-specific phosphosphatidylinositol 3-kinases (PI3K) inhibitor commonly utilized in vitro to inhibit autophagy. PI3K activity hindrance leads to inhibition of autophagosome maturation. However as different PI3K members are implicated in many cellular processes, 3-MA action is pleiotropic and not limited to autophagy inhibition. For instance, PI3K/Akt pathway inhibition may lead to diminished activity of mTORC1 and paradoxically to autophagy induction [36]. Whereas short treatment with 3-MA was shown to reduce autophagy, prolonged treatment may conversely promote autophagy in nutrient-rich conditions [36]. In addition 3-MA was shown to induce caspase-dependent cell death independently of autophagy modulating effect [121].

3-MA was shown to enhance the effect of CPT treatment in vitro in a number of cell lines [38, 40, 42, 45, 54, 56, 62–71]. Additionally, it was shown to be an effective chemosensitiser in vivo upon intraperitoneal injection in Human Salivary Adenoid Cystic Carcinoma xenograft models [69]. In contrast, little effect was found after the addition of 3-MA to CPT treated tongue squamous cell carcinoma cells. In the latter experiment ATG5 knockout was also incapable of augmenting CPT sensitivity [60].

Similarly to CQ, 3-MA/CPT co-treatment was linked to activation of caspase 3, 9 and apoptosis [45, 54, 62, 65, 66, 68, 69, 71]. Some studies linked these with mitochondrial hyperpolarization [45, 54]. However, one study in CPT resistant A2780cp ovarian carcinoma cell line found that although the addition of 3-MA to CPT increased cell death, it had no effect on apoptosis suggesting other modality of PCD to play a role [67]. Off note in the same experiment Beclin-1 (autophagy regulation protein) knockdown enhanced cisplatin-induced cell death and apoptosis. Furthermore, 3-MA was shown to further enhance expression of CPT upregulated ER-stress markers which were linked to caspase-4 and caspase-3 activation and apoptosis [66].

#### Wortmannin

Wortmannin (WT) is another nonspecific PI3Ks inhibitor. Additionally, at high concentrations, it was shown to target other vital kinases. Furthermore, WT is characterized with PI3K-inhibition profile alternative to that of 3-MA with different relative and absolute IC_50_ values for distinct PI3K family members [36].

The effectiveness of WT to potentiate CPT antineoplastic effect was shown in vitro in NSCLC and cholangiocarcinoma cell lines and in vivo in the cholangiocarcinoma xenograft model [44, 63] WT action was linked to the intrinsic apoptotic pathway. Moreover, it was suggested WT may act as a selective CPT sensitizer in rapidly proliferating cells [44].

#### mTOR/PI3K inhibitors

mTORC1 is a master regulator of cellular metabolism linking nutrient status and growth factor (GF) signaling with autophagy induction. During starvation, a decrease in mTORC1 activity leads to TFEB, ATG13 and ULK1 disinhibition which results in increased lysosomal biogenesis and autophagy initiation [36].

Wortmanin (WT) and 3-methyladeninie (3-MA) are examples of PI3K inhibitors. Additionally, WT is capable of mTOR inhibition. Due to their common utilization as autophagy inhibitors, they are discussed in other sections.

PKI-402 is an inhibitor of mTOR kinase as well as of PI3K α, β, γ and δ isoforms. It was shown to potentialize antineoplastic effect of CPT in hepatocarcinoma cell lines [72]. This was suggested to depend on lysosomal-mitochondrial crosstalk disruption. CPT alone is capable of lysosomal biogenesis induction via mtROS/TFEB pathway. Increased lysosomal activity favors mitophagy and damaged mitochondria removal thus limiting mtROS generation and conferring CPT resistance. PKI-402 addition to CPT was shown to further increase lysosomes number as well as it induced mitochondrial depolarization, mtROS generation and led to apoptosis through LMP induction. Furthermore, LMP seems to lay upstream of mtROS overproduction and apoptosis. However it is important to note that whereas low ROS accumulation may lead to compensatory lysosomal biogenesis and act protectively, high amounts may itself facilitate LMP [34]. In the same study rapamycin (mTORC1 inhibitor) was shown to confer CPT resistance probably due to autophagy and lysosomal biogenesis induction [72]. Of note, it would be beneficial to assess the influence of PKI-402 on autophagic flux which was not elucidated in the aforementioned study.

Conversely, another mTOR/PI3K inhibitor - AZD2014 was shown to antagonize CPT effect in glioblastoma, NSCL, and normal cells [44]. This discrepancy may be attributed either to cell line and condition dependence or different profile of drugs action. Although both compounds target the same enzymes, their inhibitory properties are different [122, 123].

#### MAPK inhibitors

Mitogen-activated protein kinases (MAPKs) are the group of serine/threonine protein kinases involved in extra- and intracellular signaling transduction, amplification and coordination. Classically they may be grouped in three modules forming three kinase signaling chains: (i) MAPK/ERK (Ras-Raf-MEK-ERK), (ii) JNK/p38 and (iii) MEK5/ERK5 pathways [124].

U0126 is a selective MEK1/2 (kinases upstream of ERK) inhibitor. In ovarian cancer cell lines, CPT alone was shown to activate ERK1/2, JNK, p38 and subsequently induce autophagy. Co-treatment with U0126 overcame drug resistance, reduced autophagy and promoted CPT induced apoptosis. Similar results were obtained upon 3-MA treatment as well as ERK1/2 or ATG5 knockdown. Conversely JNK and p38 inhibitors exhibited little effect [64].

U0126 was also shown to increase sensitivity towards CPT in NSCLC [73]. In this study, CPT-induced ERK-1/2 activation was linked to the upregulation of thymidylate synthase and thymidine phosphorylase which grants CPT resistance.

Inconsistently a study in the oesophageal cancer cell line showed that the addition of U0126 to CPT alleviates CPT-induced growth inhibition suppressing both senescence and apoptosis [51].

Taking into account limited research in this topic, further study is required to elucidate this discrepancy. Furthermore, it is important to note that MEK1/2 and ERK1/2 role in autophagy is complex which will be discussed later.

#### MPT0L145

MPT0L145 was designed as a selective FGF-R inhibitor and is capable of inducing non-apoptotic autophagy-dependent cell death. As it occurred MPT0L145 possesses second activity to inhibit PIK3C3 – a membrane protein implicated in endosome and autophagosome maturation process. MPT0L145 was found to reduce the viability of bladder cancer cell lines in vitro. This was associated with mitochondrial dysfunction, ROS production, and DNA damage. Moreover enhanced induction with concomitant impairment of late-stage autophagy (resulting in incomplete autophagic flux) and perinuclear accumulation of enlarged and deacidified late-endosomes were found after MPT0L145 stimulation. Whereas autophagic flux stalling and endosome maturation disruption were ascribed to PIK3C3 inhibition, simultaneous inhibition of FGF-R seems to induce autophagy potentializing cytotoxic effect. Furthermore, ATG5-knockout rescued cells from MPT0L145 induced cell death which indicates the importance of autophagic cell death in MPT0L145 cytotoxicity [74].

In the same study, MPT0L145 addition was found to markedly diminish cell viability in CPT-treated CPT-resistant bladder cancer cell line. However, the importance of autophagy was not directly evaluated for MPT0L145/CPT combined treatment [74].

#### Nimotuzumab

Nimotuzumab is an anti-EGF-R monoclonal antibody commonly utilized in cancer therapy [75].

In oesophageal squamous cell carcinoma cell line nimotuzumab was found to enhance the sensitivity towards CPT or paclitaxel-induced viability reduction. The extent of such effect positively correlated with EGF-R levels and required its high expression. Nimotuzumab alone induced autophagy which was further enhanced by the addition of CPT or paclitaxel. Notably, chemosensitising effects of nimotuzumab was abrogated by ATG-5 knock-down indicating for the importance of autophagy induction in cytotoxicity [75].

#### SAR405

PIK3C3/Vps34 (phosphatidylinositol 3-kinase, catalytic subunit type 3) is a lipid kinase localized on vesicular compartment membranes which play a vital role particularly in vesicular trafficking and autophagosome maturation. Its inhibition is linked to decreased autophagy, disruption of late endosomal compartment with the presence of swollen late endosome-lysosomes and defects in proper cathepsin D maturation [76].

SAR405 belongs to specific inhibitors of PIK3C3/Vps34. It was proved to enhance cytotoxic effect of CPT in both CPT-sensitive and resistant urothelial carcinoma cell lines [42].

#### Cepharanthine

Cepharanthine (CEP) is a cholesterol trafficking inhibitor targeting Niemann-Pick disease type C1 (NPC1) protein at lysosomal/autolysosomal membrane thus preventing cholesterol efflux into cytoplasm. It was shown to comprise lysosomal function by cholesterol accumulation, rise in luminal pH value as well as by facilitating mTORC1 dissociation from lysosomal membrane and its inactivation. Off note impaired NPC1 functioning and lysosomal cholesterol accumulation was found to disrupt autophagosome-lysosome fusion [77, 125].

CEP was found to synergize with CPT to reduce tumor growth in breast and lung cancer xenograft models. However, the specific mechanism of this effect was not evaluated [77].

#### Metformin

Metformin, a biguanid derivative with antihyperglycemic activity, is the most widely used orally administered antidiabetic drug. The molecular mechanism of metformin is versatile. Metformin inhibits mitochondrial respiratory chain (complex I) and mitochondrial glycerophosphate dehydrogenase. Moreover, it was suggested to affect the lysosomal membrane to favor mTORC1 dissociation from the regulatory complex and its deactivation. The aforementioned effects result in increased AMP:ATP ratio, AMPK activity and resultant changes in cellular metabolism including: enhanced B-oxidation, glucose uptake, glycolysis, mitophagy and autophagy [78]. Importantly some of the aforementioned mechanisms were proved for millimolar concentrations of metformin. Moreover, large cohort study inked metformin to decreased cancer burden. Particularly metformin was showed to improve neo-adjuvant therapy outcomes in neck, cervix and breast cancer. Consistently in vitro experiments confirms metformin antineoplastic activity [79].

Saladini et al. showed that metformin in micromolar (5–30 μM) concentrations effectively sensitized breast and cervical cancer cell lines towards CPT. This was associated with a decrease in CPT-induced autophagy and increased apoptosis. Metformin alone was also capable of triggering those changes and inhibited cell proliferation, however to a lesser extent. The action of micromolar concentrations of metformin was independent of ATP production alteration and AMPK/mTOR pathway. On the other hand, metformin treatment led to a decrease in cellular ammonia production which was attributed to direct inhibition of glutaminase (GLS) [79].

GLS is an enzyme responsible for glutamine deamination and ammonia production. It is often overexpressed in cancer which may utilize glutamine as an additional source of energy and nitrogen for incorporation into amino acids. Moreover ammonia accumulation was shown to induce autophagy in a non-canonical mTORC1 independent manner. Consistently metformin in micromolar concentrations diminished ammonia production as well as it reduced MAP1LC3B-II, GABARAP, BECN1 and ATG12/ATG5 expression. GLS-silencing reproduced metformin effects. Furthermore, CPT and metformin co-treatment lead to further decrease in ammonia concentration supporting the contribution of this mechanism to CPT-chemosensitization. Interestingly metformin increased BCL2-BECN1 binding thus sequestering the first and possibly preventing its antiapoptotic action [79].

#### 2-Deoxy-D-glucose

Cancer cells may depend on glycolysis as a primary source of ATP. This widely studied metabolic shift, often considered as one of cancer hallmarks, is called Warburg effect. Energetic imbalance with alerted ATP:ADP ratio leads to changes in autophagy and cell death which may potentialize antineoplastic efficacy of chemotherapeutics [80].

In this context 2-Deoxy-D-glucose (2-DG) – glycolysis inhibitor, was evaluated as a CPT-sensitizer in cancer. 2-DG treatment alone was showed to induce apoptosis in RKO colon carcinoma cells and neuroblastoma cell lines (Tet21N, SK-N-BE(2), SH-SY5Y). On the other hand, it attenuated apoptosis in HCT116 colon carcinoma cell line suggesting its effect to be cell line-specific. 2-DG triggered endoplasmic reticulum (ER) stress, suppression of which alleviates apoptosis in SK-N-BE(2). Importantly 2-DG induced ER-stress facilitates autophagy induction in HCT116 but not in SK-N-BE(2). Consistently ER-stress or autophagy inhibition with Baf-A1 diminished the protective role of 2-DG in HCT116. 2-DG additively enhanced apoptosis in CPT-treated SK-N-BE(2) which was diminished by ER-stress inhibition or autophagy induction by rapamycin. In contrast, 2-DG rescued HCT116 from CPT-induced apoptosis which was even more visible after the addition of rapamycin and attenuated by ER-stress inhibition [80].

This suggests glycolysis inhibition by 2-DG leads to ER-stress and its effect on cell growth depends on the direction of subsequent changes in autophagic flux. Furthermore, 2-DG may play a dual role as an adjuvant for CPT treatment either promoting or diminishing antineoplastic activity depending on cell line.

Another study assessed the combination of CPT and 2-DG in glioma grade IV cell lines. The combination of drugs led to synergistic reduction of cell viability and induction of apoptosis both under normoxia and hypoxia conditions. 2-DG alone was shown to induce autophagy which was probably attributed to increased ER-stress. Interestingly CPT alone decreased autophagy in those cell lines standing in contrast with other studies in which CPT mediated autophagy induction was emphasized. Even more strikingly co-treatment with CPT and 2-DG led to the reduction of both ER-stress (measured by BIP expression) and autophagy. Of note the this effect was replicated by co-treatment with 2-DG and Akt inhibitor LY294002 [81].

The aforementioned studies highlight the importance of autophagy in 2-DG–CPT interplay in cancer cells. However, owing cell line specificity of the co-treatment effect and some inconsistent results further study is required to elucidate the role of 2-DG in CPT-chemosensitization.

#### Insulin

Insulin is a peptide hormone secreted by the pancreas implicated in glucose cellular uptake and metabolism regulation in general. Due to its pleiotropic activity, insulin is involved in many pathologies including cancer disease. Insulin membrane receptor is a tyrosine kinase receptor activation of which facilitates Akt and MAPK/ERK signaling [82].

In the oesophageal squamous cell carcinoma cell line insulin and CPT co-treatment was showed to increase Akt and mTOR expression and decrease autophagy initiation which correlated with increased apoptotic ratio [82].

#### Nicotinamide Phosphoribosyltransferase inhibitor

Nicotinamide Phosphoribosyltransferase (Nampt) is a rate-limiting enzyme catalyzing conversion of nicotinamide to nicotinamide mononucleotide as an initial step of nicotinamide adenine dinucleotide (NAD+) salvage pathway. NAD is a substrate in many cellular synthetic pathways as well as a vital signaling molecule. Therefore Nampt up-regulation found in several tumors may be an important event in carcinogenesis [83].

FK866 is a specific Nampt inhibitor. It was shown to induce autophagy-dependent cell death in neuroblastoma and cervical cancer cell lines. Its cytotoxicity was reduced by 3-MA and potentiated by CQ indicating the importance of early-stage but not late-stage phases of autophagy in the process. Co-treatment with CPT or etoposide and ineffective concentrations of FK866 was found to synergistically induce cell death in neuroblastoma and cervical cancer cell lines. FK866 addition to CPT or etoposide was suggested to unmask mitochondrial NAD depletion. However, no effect of autophagy inhibition on CPT/FK866 co-treatment was evaluated [83].

#### Oridonin

Oridonin is a naturally occurring plant terpenoid extensively studied in terms of cancer chemosensitisation i.e. towards CPT. It was shown to induce apoptosis in cancer through p-AMPK degradation dependent glucose/lactate metabolism imbalance and consequent autophagy activation [126]. Oridonin alone had little effect on cell viability in ovarian cancer cell lines. However, administration of oridonin to CPT-treated cells led to a great increase in apoptosis even in CPT-resistant cell line. This was associated with Belin-1 downregulation and autophagy reduction. Importantly 3-MA addition further increased cell death and rapamycin (autophagy inducer) or Beclin-1 overexpression antagonize oridonin effects. Off note oridonin showed no effect in CPT treated normal epithelial ovarian cell line suggesting its effect to be cancer-specific [127]. Another study proved oridonin mediated CPT sensitization to occur in NSCLC cell line [128]. Here oridonin treatment was linked to AMPK/Akt/mTOR-dependent autophagosome accumulation and apoptosis. Subsequently, it was shown to protect mice from CPT-induced nephrotoxicity.

#### Phenoxofiol

Phenoxofiol (PXD) is an isoflavone analog with widely proved anticancer activity. It was approved by FDA for clinical studies, however, no statistically significant beneficial outcome of treatment was reported. The molecular mechanism of PXD action was suggested to involve X-linked inhibitor of apoptosis protein (XIAP) and FLICE inhibitory protein (FLIP) downregulation as well as direct inhibition of topoisomerase II and ENOX2 (ecto-NOX disulfide-thiol exchanger 2). PXD was shown to sensitize ovarian cancer cell line towards CPT [47, 85]. This was accompanied by XIAP downregulation and autophagy inhibition with Beclin-1 inhibition. Interestingly siRNA knockdown of XIAP was shown to conversely enhance autophagy in response to CPT and was less efficient in sensitizing cells towards drug-induced growth inhibition. This suggests PXD chemosensitising action depends on double activity to simultaneously diminish XIAP level and autophagy [47].

In addition, PXD conferred protection against CPT-induced neurotoxicity in vitro proving it may simultaneously limit adverse effects of therapy [86]. Of note, another study provides evidence for PXD efficacy in carboplatin chemosensitisation in vitro and in vivo in ovarian cancer [86].

#### Andrographolide

Andrographolide is a naturally occurring labdane diterpenoid. It was characterized by various biological activities including anti-inflammatory, antiviral, antioxidant and anticancer action. Molecularly andrographolide effects were mainly attributed to Nrf2 activation which in turn facilitates oxidative stress response thus limiting ROS accumulation. Additionally, andrographolide was shown to disrupt endocytic receptor degradation by affecting trafficking from late endosome to lysosomes [87].

Andrographolide was shown to sensitize NSCLC and colon cancer cell lines in vitro towards CPT induced growth suppression and apoptosis [87–89] Moreover it improved CPT therapy outcomes in NSCLC xenograft model including lifespan prolongation [89]. These actions were linked to andrographolide capacity to inhibit autophagic flux. In one study the latter was attributed to PTEN downregulation, consequent Akt and mTORC1 activation. Both wortmannin (used as Akt inhibitor) and vector-mediated PTEN overexpression was shown to attenuate CPT and andrographolide co-treatment efficacy [88]. However these results require cautious interpretation as both wortmannin and PTEN transfection may act on various pathways. Other studies indicated that androghapholide CPT-sensitizing activity may result from its ability to disrupt autophagosome-lysosome fusion [87, 89]. Off note andrographolide did not affect other lysosomal functions [87]. Moreover silencing Beclin-1 or ATG-7 impairs andrographolide activity suggesting that its activity depends on late flux stalling rather than complete autophagy inhibition [87, 89] The latter also indicates andrographolide may be more efficient in cells with a higher level of autophagy for instance due to CPT stimulation. Off note in the same study ATG-7 knock-down itself sensitizes cells towards CPT [87].

#### 4-Acetylantroquinonol

Antroquinol is a tetrahydro ubiquinone derivative found in Antrodia camphorate, a type of mushroom popular in Taiwan and Chinese medicine, reported to possess anticancer activity [46].

Synthetic derivative of antroquinol - 4-acetylantroquinonol (4-AAQB) was shown to synergistically induce apoptosis with CPT in ovarian cancer cell lines. Moreover, the addition of 4-AAQB to CPT regiment reduced tumor growth in ovarian cancer xenograft models after oral or intraperitoneal administration. The compound was shown to reduce autophagy in CPT-treated cells as measured by Atg-5 and Atg-7 downregulation. Simultaneously 4-AAQB was shown to reduce Akt and mTOR phosphorylation which is known to induce rather than reduce autophagy yet the opposite effect was noted [46].

#### Pristimerin

Pristimerin is a quinonemethide triterpenoid with anti-cancer activity isolated from Celastraceae and Hippocrateaceae. It was shown to affect apoptosis, autophagy and drug resistance. Molecularly pristimerin was shown to directly inhibit proteasome and telomerase activity, to diminish MEK/ERK, EGF-R, PI3K/Akt, Wnt/β-catenin, NfxB pathways as well as to induce JNK [90, 91].

The effect of pristimerin on autophagy is not clear. Some studies showed it to reduce autophagy initiation whereas others indicated for facilitating incomplete autophagic response with cytoplasmic vacuolization and accumulation of autophagy-related proteins [90–92] Moreover one study showed that autophagy initiation inhibition by 3-MA reduced pristimerin cytotoxicity indicating for the role of autophagy-dependent cell death [92]. Interestingly the same study subsequently found that ERK1/2 inhibition was required for pristimerin/paclitaxel co-treatment mediated LC3-II accumulation. That stands in opposition to the majority of studies suggesting that ERK1/2 induces autophagy.

Of note MEK/ERK pathway was shown to interact with autophagic machinery at multiple levels. MEK/ERK is required for maintaining a basal level of autophagy which depends on direct interaction with TSC [129, 130] This is necessary for proper TSC activity and mTORC1 inhibition which promotes autophagy initiation. Conversely sustained activation of ERK seems to diminish autophagy [117]. Moreover, MEK inhibition was suggested to alert irradiation-induced autophagy at late stages [129]. Of note MEK can bypass ERK to promote autophagy. On the other hand, LC3-II availability on autophagosomes seems to be necessary with ERK1/2 activity [117].

Nevertheless in each case, pristimerin reduced cancer viability. In terms of CPT antineoplastic activity, pristimerin was shown to synergize with the drug in NSCLC cell line in vitro and in xenograft experiment. This was linked with downregulation of miR-23a, reduction of Akt and GSK3B phosphorylation and inhibition of autophagy initiation [91].

#### Icariin

Icariin is a flavonoid usually derived from Epimedium sagittatum with various biological effects including anti-cancer, anti-inflammatory, anti-oxiant, anti-apoptotic and anti-autophagic activity. Icariin was showed to sensitize multidrug-resistant ovarian cancer cell line towards CPT mediated apoptosis in vitro. It was linked with reduced autophagic flux. Furthermore, autophagy induction by rapamycin or Atg-5 overexpression partially diminished CPT-icariin co-treatment mediated cytotoxicity [93].

#### Melatonin

Melatonin is a hormone secreted by pineal glands and regulating the sleep-wake cycle. It is also an anti-oxidant capable of both direct free radicals scavenging and Nrf-2 induction. The latter is also linked to autophagy induction. Interestingly melatonin was shown to differentially affect autophagic response upon hypoxia/reoxygenation induced oxidative stress - promoting it in normal cells thus acting pro-survival while reducing autophagy in cancer thereby facilitating apoptosis [94].

Co-treatment with CPT and melatonin was showed to potentiate apoptotic response in cervical and head and neck squamous cell carcinoma [95, 96] It was shown to increase the pool of damaged mitochondria and mitochondrial ROS production which enhanced CPT mediated apoptosis. This effect may be attributed to melatonin triggered alteration of mitophagy which normally has a protective role against CPT induced mitochondrial damage. Whereas CPT was shown to induce JNK/parkinin pathway thus promoting mitophagy, the addition of melatonin decreased JNK activation and mitochondrial removal. Of note aforementioned studies reported contradictory data regarding conventional autophagy as the autophagy-related genes were found to be either up- or down-regulated after the addition of melatonin.

#### Procyanidins

Procyanidins (OCP) are members of flavonoids suggested possessing anti-oxidative, anti-inflammatory, antimicrobial, antiviral and anti-cancer activity [97].

OCP was shown to significantly enhance CPT-induced apoptosis while simultaneously inducing autophagy. Importantly pre-treatment with 3-MA to reduce autophagy was shown to decrease pro-apoptotic activity implying autophagy involvement in cell death [97].

#### Neferine

Neferine is a bisbenzylisoquinoline alkaloid with anti-cancer activity derived from Lotus seeds. This was showed to depend on P-glycoprotein 1 (P-gp) direct inhibition and/or downregulation. P-gp is a member of the ATP-binding cassette sub-family B (ABCB) transporter family. It is often upregulated in cancer-promoting drug efflux and multidrug-resistant phenotype. Interestingly neferine preferentially reduces the viability of drug-resistant cell lines [98].

Nerefin co-treatment effectively enhanced CPT mediated cell viability reduction in NSCLC cell line in vitro. This was associated with increased ROS generation and downregulation of PI3K/Akt/mTOR pathway. Furthermore, induction of autophagy observed as an increased LC3B-II/LC3B-I ratio was reported. Autophagic flux was possibly stalled at a late stage which was the cause of acid vesicular accumulation. The involvement of non-canonical autophagy rather than canonical was proposed as neferine treatment subsequently lead to the downregulation of Beclin-1 and PI3KCIII. Additionally ROS scavenger – glutathione was suggested to reverse neferine effects on autophagy and cell viability. Interestingly pre-treatment with 10uM CQ partially reversed neferine/CPT viability loss, reduced AV accumulation and decreased LC3B-II/LC3B-I ratio which is somehow contradictory taking into account CQ molecular mechanism. Of note it was reported that pre-treatment with higher CQ concentrations further reduced the viability of neferine/CPT treated cells [99].

#### Quercetin-3-O-β-D-galactopyranoside (hyperoside)

Hyperoside is a flavonol glycoside present mainly in members of Hypericum and Crataegus genera. It was characterised with anti-inflammatory, anti-oxidant and anticancer activities [100].

Hyperoside was shown to synergistically induce apoptosis with CPT in the ovarian cancer cell line. This was associated with increased autophagy. Its inhibition with 3-MA attenuated the effect suggesting the role of autophagic cell death. Additionally decreased phosphorylation of Akt and Bcl-2 expression were involved. Interestingly the magnitude of hyperoside effect correlated with progesterone receptor membrane component 1 (PGRMC1) expression. PGRMC1 is often upregulated in drug-resistant cancer cells which is linked to protective autophagy, cytochrome p450 activation and pro-survival advantage. Moreover PGRMC1 is able to bind with LC3B-II and is implicated lysosome-autophagosome function as well as essential for the proper course of autophagy [100]. These results suggest hyperoside may act as CPT sensitizer with selective action towards CPT-resistant cells characterized with high PGRMC1 expression and autophagic flux.

#### Bu-Zhong-Yi-Qi decoction

Bu-Zhong-Yi-Qi Decoction (BZYQD) is a traditional Chinese herbal medication comprising extracts from six different herbs. It was shown to induce apoptosis in some cancer cell lines [101].

BZYQD was shown to sensitize resistant NSCLC cell line towards CPT mediated apoptosis. This was associated with increased autophagy induction and accumulation of LC3-II and Atg7. Importantly 3-MA addition significantly reduced BZYQD/CPT cytotoxicity which suggests the involvement of autophagic cell death. Moreover, co-treatment increased ROS generation and scavenging them with N-acetylcysteine almost completely suppressed cell death [101].

#### Monanchocidin A

Monanchocidin A (MonA) is an alkaloid isolated from marine sponge Monanchora pulchra exhibiting cytotoxic properties in cancer cell lines [102].

MonA in combination with CPT was showed to synergistically induce cell death in CPT-resistant germ cell tumor cell line. Moreover, MonA alone was equally cytotoxic against CPT-sensitive and resistant cell lines of germ cell tumor, prostate and bladder cancer, whereas non-malignant cells were less sensitive for MonA induced cell. Long-term low-doses (<2uM) MonA treatment was associated with unselective autophagic protein degradation which was blocked by 3-MA treatment. Interestingly high-dose (>2uM) MonA induced rapid and unspecific degradation of proteins was not inhibited by 3-MA. Furthermore, high concentrations of MonA were shown to promote LMP. It was proposed that MonA has biphasic dose-dependent action. At lower doses autophagy induction is a predominant mode of action whereas higher doses preferentially facilitate LMP [102].

#### (−)-Epigallocatechin gallate

(−)-Epigallocatechin gallate (EGCG) is the main member of polyphenolic catechin present in green tea with numerous studies exploring its anti-cancer activity [103].

The synergistic effect of EGCG and CPT or oxaliplatin treatment on proliferation reduction and cell death in colorectal cancer cell lines was explored. This was associated with increased autophagosome formation and accumulation. Importantly siRNA knock-down of autophagy-related ATG genes was shown to reverse EGCG chemosensitising effect which suggests the involvement of autophagic cell death [103].

#### Chalcone-24

Chalcone-24 (Chal-24) is a member of chalconoids – a group of natural phenols with anti-microbial, anti-inflammatory, and anti-neoplastic activity. Chal-24 efficiently induces cell death in a cancer cell in vitro and in vivo without signs of toxicity in mice. Moreover, it was shown to facilitate autophagy-dependent necroptosis [104].

Chal-24 was found to synergistically induce apoptosis with CPT in NSCLC cell lines. CPT/Chal-24 co-treatment led to an increase in JNK/Bcl-2/Beclin 1 dependent autophagy induction, facilitated ERK-dependent proteasomal degradation of cellular inhibitor of apoptosis proteins (c-IAPs) and triggered Ripoptosome (RIP1/FADD/caspase 8) complex assembly as well as it dramatically reduced levels of Riopotosome inhibitor – cFLIP_L_. Importantly autophagy inhibitor (CQ, WT or 3-MA) addition partially reversed CPT/Chal-24 toxicity suggesting the importance of the autophagic process in cell death [104].

#### Resveratrol

Resveratrol is a natural polyphenolic compound associated with pleiotropic beneficial activities in human including anti-neoplastic effect. Resveratrol molecular mechanism was linked to many molecular pathways and its potential ability to directly influence cell membrane was highlighted [105, 106].

Resveratrol was shown to synergistically induce apoptosis with CPT in NSCLC cell line. This was associated with autophagic flux induction. Furthermore, its inhibition by 3-MA reduced apoptotic response. Moreover combined treatment led to an increase in Bax expression and subsequent decrease in Bcl-2 and Akt phosphorylation [107].

#### Gambogic acid

Gambogic Acid (GA) is a xanthonoid compound derived from Garcinia hanburyi with anti-cancer activity proved in vitro and in vivo [108].

Co-treatment with CPT and GA was shown to subsequently enhance growth inhibition and autophagy of NSCLC cell lines compared to monotherapy. The effect on cell death could be markedly reduced by the addition of autophagy inhibitors – 3-MA and CQ. Moreover, treatment with GA was linked to Akt/mTOR pathway inhibition and the addition of mTOR inhibitor - rapamycin further increased autophagic flux and cell death [108].

#### Fungal immunomodulatory proteins

GMI is an immunomodulatory protein derived from Ganoderma microsporum fungus. It was shown to facilitate telomerase inhibition, senescence and autophagic cell death in cancer cells. Moreover its antineoplastic activity was proved in vivo after oral administration. GMI activity was attributed to autophagic cell death characterized with unfused autophagosome accumulation which depended on ER stress/calcium/Akt/mTOR pathway [109].

In NSCLC CPT and GMI co-treatment was shown to synergistically induce apoptosis. Importantly while 3-MA or LC3 downregulation led to the reduction of GMI/CPT induced apoptosis, CQ addition further increased enhanced apoptotic response. This suggests an early stage but not late-stage autophagy is important in GMI action. Furthermore, GMI promoted the downregulation of ERCC1, XPF, and survivin [109].

#### Glaucocalyxin B

Glaucocalyxin B (GLB) is a diterpenoid with anti-cancer activity extracted from Rabdosia japonica [110].

Low and non-toxic doses of GLB were found to sensitize gastric cancer cell line towards CPT induced cytotoxicity. This was accompanied by enhancement of DNA damage, ROS production and greatly increased autophagy. However, no further experiments were performed to elucidate the role of autophagy in GLB/CPT cytotoxicity [110].

#### Poly-unsaturated fatty acids

Poly-unsaturated fatty acids (PUFAs) positive role in human organism is nowadays widely highlighted [131].

Arachidonic acid (AA), eicosapentaenoic acid (EPA), docosahexaenoic acid (DHA) were shown to enhance CPT mediated viability reduction in NSCLC cell line in vitro. The effect was more pronounced for EPA and DHA. Additionally EPA and DHA but not AA co-treatment with CPT was linked with higher caspase 3/7 activity and an increase in autophagy. Importantly autophagy inhibition by 3-MA reversed DHA and EPA induced sensitization indicating for the importance of autophagic cell death in this process [111].

Consistently in cervical cancer cell line oleanolic acid (OA) was shown to enhance CPT mediated viability reduction. Interestingly this was associated with reduced caspase 3 and PARP cleavage, but with Bcl-2 downregulation and increased LC3B-II expression. This indicates that autophagic cell death may be the main mechanism CPT-sensitization in the case of OA. Strikingly this study simultaneously proved that OA treatment was effective in limiting CPT-mediated nephrotoxicity in mice which correlated with the reduction of: oxidative stress, apoptosis, autophagy, NfxB, STAT3 and Erk1/2 signaling [112]. These results are of particular interest as OA seems to exert the opposite effect on autophagy in kidney tissue and cancer cells.

#### Quinacrine

Quinacrine (QC) is an anti-malarial drug structurally related to CQ.

QC was shown to synergize CPT or carboplatin anti-proliferative activity in ovarian cancer and head and neck squamous cell carcinoma cell lines [113, 114] Importantly chemoresistant cell lines were more prone for QC/CPT co-treatment effect. QC promoted autophagic flux as well as autophagosome accumulation. The addition of Baf-A1 reversed QC mediated cell death and chemosensitisation. Interestingly the reduction of p62 linked to increased autophagic flux was suggested to play a role in the aforementioned actions as p62 knock-down reproduced QC effect. p62 is an autophagosome protein interacting with LC3 and acting as a cargo receptor. It is necessary for selective autophagy. Moreover, combinations of QC and carboplatin or CPT were shown to reduce tumor growth respectively in chemoresistant ovarian and head and neck squamous cancer xenograft model more efficiently than monotherapy [113, 114].

#### Graphene oxide

In recent years graphene gained extensive attention in terms of medical nanotechnology, particularly drug delivery systems. Graphene oxide (GO) is a single-atomic layered chemically modified graphene containing various oxygen functional groups. GO is characterized by great intracellular penetration. Clathrin-mediated endocytosis is believed to be one of the possible routes for GO enter. GO was showed to colocalize with lysosomes which further supports this vision. Interestingly GO was also showed to localize in cytoplasm and nucleus which was attributed to lysosomal escape. Only high concentrations of GO were shown to disrupt lysosomal membrane stability triggering lysosome-dependent cell death. Importantly GO subsequently induces autophagy and blocks its flux at late stages due to low degradative activity of lysosomes [115]. Interestingly in nuclei GO was shown to colocalize with LC-3. Furthermore, knock-down of ULK1 or Atg-7 but not inhibition of autophagosome-lysosome fusion comprises LC-3/GO nuclear localization suggesting autophagosome formation and elongation are required for GO nuclear trafficking [116]. Off note LC-3 nuclear pool is also present physiologically and important in autophagy regulation [132].

Treatment with GO/CPT formulation markedly enhanced cell death in cervical, prostate, ovarian, and colon cancer cell lines compared to CPT alone. However, no significant difference was found in A549 NSCLC cells. GO/CPT was shown to induce necrosis whereas apoptotic response was not significantly affected. Consistently GO/CPT treatment facilitates autophagy initiation in all researched cell lines apart from A549 and autophagy induction was required for CPT/GO/LC-3 nuclear localization as well as for necrotic death [116].

#### Proteasome inhibitors

The proteasome is a protein enzymatic complex with proteolytic activity implicated in the degradation of proteins tagged with ubiquitin. This process is implicated in the proper regulation of cellular pathways as well as important in oxidative and unfolded protein stress response. Due to the accumulation of damaged protein characteristic for some cancers proteasome inhibitors as bortezomib are successfully utilized in chemotherapy regimens [133].

Bortezomib was found to block autophagic flux without affecting autophagosome-lysosome function. This was linked to the suppression of lysosomal cathepsin activity which depended on sustained ERK phosphorylation. Moreover, bortezomib co-administration synergized with the cytotoxic effect of CPT in ovarian cancer [117].

#### Zoleandronic acid

Bisphosphonates, with zoleandronic acid (ZA) being one of them, are a group of drugs that affect bone tissue metabolism. ZA is utilized in the prevention and treatment of multiple bone disorders as osteoporosis and bone metastasis. Importantly its administration was linked to improve survival in cancer patients which was attributed to reduced incidence of bone metastasis [118].

Co-treatment with ZA and CPT or paclitaxel was shown to synergistically facilitate apoptotic response in a salivary adenoid cystic carcinoma cell line in vitro. This was accompanied by increased ROS accumulation and autophagy marker - LC3B expression. Furthermore, ROS scavenging with N-acetylcysteine, autophagy inhibition by Beclin-1 knockdown or pre-treatment with 3-MA reduced ZA monotherapy effect on apoptosis. However, effects of these manipulations on ZA/CPT co-treatment were not evaluated [118].

#### Lithium

Lithium (Li) is known to induce autophagy. In oesophageal and colorectal cancer cells Li treatment led to ineffective autophagy induction that was stalled at late-stages and caused peripheral cytoplasmic accumulation of vesicles. Consistently CQ addition led to a mild further increase in vesicles accumulation in Li-treated cells in contrast to a marked effect in rapamycin-induced autophagy. Li was shown to chemosensitise cancer cells towards CPT or 5-FU which was also accompanied by enhanced accumulation of cytoplasmic vesicles. This effect was not replicated by 5-FU/rapamycin/CQ or Baf-A1 co-treatment suggesting that additional Li effects, apart from late-autophagic event inhibition, may play a role. Furthermore, Li induced depletion of LAMP-1, LAMP-2 and cathepsin B indicating for comprised lysosomal stability. At last Li with oxaliplatin was found to synergistically inhibit tumor growth in the xenograft colorectal carcinoma model [119].

#### C60(Nd) nanoparticles

C60(Nd) nanoparticles (C60(Nd)) are fullerene (allotrope of carbon) derivative with neodymium (Nd) atom trapped inside a spherical “cage” made of carbon atoms. Their intracellular trafficking depends on endocytosis, pinocytosis and passive membrane permeation. C60 were shown to induce cytotoxic autophagy and to sensitize cancer cells towards chemotherapeutics [120, 134].

Pre-treatment of cervical cancer cell lines with non-toxic concentrations of C60(Nd) was showed to greatly potentiate CPT mediated cell death. The same effect was achieved concerning doxorubicin. Unfortunately, precise mechanism of C60(Nd) action was elucidated for doxorubicin (DOX) co-treatment and the following conclusions came from those experiments. The addition of C60(Nd) to DOX resulted in enhanced autophagy and its inhibition by 3-MA or WT partially reduced the chemosensitising effect. Moreover, co-treatment led to increased ROS accumulation, scavenging of which with N-acetylcysteine rescued cells from both autophagy induction and chemosensitisation. Conversely, autophagy induction by rapamycin was showed to alleviate DOX cytotoxicity [120]. These results suggest that C60(Nd) mediated chemosensitisation depends on ROS induced cytotoxic autophagy.

## Conclusion

Approach to alleviate cisplatin resistance by compounds that affect autophagy and/or lysosomes is justified by the great potential of the drug to induce both. Two different strategies may be utilized. First, inhibition of autophagy and lysosome biogenesis may diminish their protective role. On the other hand, successful effect may be achieved by modulating the physiology of autophagosomes and/or lysosomes to uncover their death-promoting functions.

Inhibition of lysosomes biogenesis would restrict vesicular cisplatin accumulation and its efflux via exocytosis/extracellular vesicles. Furthermore, autophagy inhibition would limit its protective role manifested in its antiapoptotic and pro-survival functions. The aforementioned research supports this concept – some natural or synthetic substances were shown to limit autophagy induction and alleviate cisplatin resistance. Moreover, results obtained from the knockout or downregulation of autophagy-related genes further confirms this notion.

Cisplatin is characterized with a great potential to induce autophagy and cisplatin-resistant cells to present both high level of basal autophagy and alterations in vesicular [14–18]. The latter includes elevated extracellular vesicles secretion, alteration in lysosomal size, number and localization as well as disrupted localization and functioning of endosomal recycling compartment. Consequently, CPT treatment-induced autophagy shown to facilitate drug resistance was also suggested to confer autophagy induction vulnerability. CPT-resistant cells with a higher level of basal autophagy were much more prone to undergo apoptosis after glutamine deprivation than CPT-sensitive cells [135]. This depended on AMPK/ULK1-triggered autophagy. It was also suggested that ULK-1-triggered autophagy (especially mitophagy) leads to ROS overproduction which may contribute to cells entry into apoptosis.

Furthermore, cisplatin may affect lysosomal stability both directly (f.i. affecting membrane fluidity) and indirectly by increased ROS production, decreased LAMP-1 and 2 expressions [14–18]. This convergence of different molecular events characteristic for cancer and further augmented by cisplatin/cisplatin resistance on lysosomes makes them a potential target for cancer therapy. Consistently autophagy late-stage inhibition in cells with a high level of autophagy, facilitating excessive induction of autophagy, promotion of LMP and others not yet elucidated mechanisms were shown to sensitize cancer cells to cisplatin.

The search for novel substances for cancer therapy targeting the aforementioned mechanisms is encouraged by a recent report of tacrine-melatonin heterodimer C10 antineoplastic activity [136]. This compound induces senescence and apoptosis of cancer cells which is dependent on its double activity: as autophagy inducer and late-stage autophagy inhibitor.

### Data availability (data transparency)

not applicable.

## References

[1] Ghosh S (2019). Cisplatin: the first metal based anticancer drug. Bioorg Chem.

[2] Makovec T (2019). Cisplatin and beyond: molecular mechanisms of action and drug resistance development in cancer chemotherapy. Radiol Oncol.

[3] Eljack ND, Ma HYM, Drucker J, Shen C, Hambley TW, New EJ, Friedrich T, Clarke RJ (2014). Mechanisms of cell uptake and toxicity of the anticancer drug cisplatin. Metallomics.

[4] Florea AM, Büsselberg D (2011). Cisplatin as an anti-tumor drug: cellular mechanisms of activity, drug resistance and induced side effects. Cancers (Basel).

[5] Arnesano F, Losacco M, Natile G (2013). An updated view of Cisplatin transport. Eur J Inorg Chem.

[6] Safaei R, Katano K, Larson BJ (2005). Intracellular localization and trafficking of fluorescein-labeled Cisplatin in human ovarian carcinoma cells - PubMed. Clin Cancer Res.

[7] Chen SH, Chang JY (2019). New insights into mechanisms of cisplatin resistance: from tumor cell to microenvironment. Int J Mol Sci.

[8] Nonnekens J, Hoeijmakers JH (2017). After surviving cancer, what about late life effects of the cure?. EMBO Mol Med.

[9] Dasari S, Tchounwou PB (2014). Cisplatin in cancer therapy: molecular mechanisms of action. Eur J Pharmacol.

[10] Shen DW, Pouliot LM, Hall MD, Gottesman MM (2012). Cisplatin resistance: a cellular self-defense mechanism resulting from multiple epigenetic and genetic changes. Pharmacol Rev.

[11] Martin LP, Hamilton TC, Schilder RJ (2008). Platinum resistance: the role of DNA repair pathways. Clin Cancer Res.

[12] Yamanaka K, Chatterjee N, Hemann MT, Walker GC (2017). Inhibition of mutagenic translesion synthesis: a possible strategy for improving chemotherapy?. PLoS Genet.

[13] Matsunaga T, Hojo A, Yamane Y, Endo S, el-Kabbani O, Hara A (2013). Pathophysiological roles of aldo-keto reductases (AKR1C1 and AKR1C3) in development of cisplatin resistance in human colon cancers. In: Chemico-biological interactions. Chem Biol Interact.

[14] Yun C, Lee S (2018). The roles of autophagy in Cancer. Int J Mol Sci.

[15] Safaei R, Larson BJ, Cheng TC, Gibson MA, Otani S, Naerdemann W, Howell SB (2005). Abnormal lysosomal trafficking and enhanced exosomal export of cisplatin in drug-resistant human ovarian carcinoma cells. Mol Cancer Ther.

[16] Guerra F, Paiano A, Migoni D, Girolimetti G, Perrone AM, de Iaco P, Fanizzi FP, Gasparre G, Bucci C (2019). Modulation of RAB7A protein expression determines resistance to cisplatin through late endocytic pathway impairment and extracellular vesicular secretion. Cancers (Basel).

[17] Chauhan SS, Liang XJ, Su AW, Pai-Panandiker A, Shen DW, Hanover JA, Gottesman MM (2003). Reduced endocytosis and altered lysosome function in cisplatin-resistant cell lines. Br J Cancer.

[18] Colacurcio DJ, Nixon RA (2016). Disorders of lysosomal acidification—the emerging role of v-ATPase in aging and neurodegenerative disease. Ageing Res Rev.

[19] Samuel P, Mulcahy LA, Furlong F, McCarthy HO, Brooks SA, Fabbri M, Pink RC, Carter DRF (2018). Cisplatin induces the release of extracellular vesicles from ovarian cancer cells that can induce invasiveness and drug resistance in bystander cells. Philos Trans R Soc B Biol Sci.

[20] Liang XJ, Mukherjee S, Shen DW, Maxfield FR, Gottesman MM (2006). Endocytic recycling compartments altered in cisplatin-resistant cancer cells. Cancer Res.

[21] Li X, Zhou Y, Li Y, Yang L, Ma Y, Peng X, Yang S, Liu J, Li H (2019). Autophagy: a novel mechanism of chemoresistance in cancers. Biomed Pharmacother.

[22] Metaxakis A, Ploumi C, Tavernarakis N (2018). Autophagy in Age-Associated Neurodegeneration Cells.

[23] Yu L, Chen Y, Tooze SA (2018). Autophagy pathway: cellular and molecular mechanisms. Autophagy.

[24] Dikic I, Elazar Z (2018). Mechanism and medical implications of mammalian autophagy. Nat. Rev. Mol. Cell Biol..

[25] Fennelly C, Amaravadi RK (2017). Lysosomal biology in cancer. Methods Mol Biol.

[26] Settembre C, Fraldi A, Medina DL, Ballabio A (2013). Signals from the lysosome: a control Centre for cellular clearance and energy metabolism. Nat. Rev. Mol. Cell Biol..

[27] Zhitomirsky B, Assaraf YG (2017). Lysosomal accumulation of anticancer drugs triggers lysosomal exocytosis. Oncotarget.

[28] Lawrence RE, Zoncu R (2019). The lysosome as a cellular Centre for signalling, metabolism and quality control. Nat Cell Biol.

[29] Mariño G, Niso-Santano M, Baehrecke EH, Kroemer G (2014). Self-consumption: the interplay of autophagy and apoptosis. Nat Rev Mol Cell Biol.

[30] Fitzwalter BE, Towers CG, Sullivan KD (2018). Autophagy inhibition mediates apoptosis sensitization in Cancer therapy by relieving FOXO3a turnover. Dev Cell.

[31] Doherty J, Baehrecke EH (2018). Life, death and autophagy. Nat Cell Biol.

[32] Bialik S, Dasari SK, Kimchi A (2018). Autophagy-dependent cell death - where, how and why a cell eats itself to death. J. Cell Sci..

[33] Wang F, Gómez-Sintes R, Boya P (2018). Lysosomal membrane permeabilization and cell death. Traffic.

[34] Yu F, Chen Z, Wang B, Jin Z, Hou Y, Ma S, Liu X (2016). The role of lysosome in cell death regulation. Tumor Biol.

[35] Aits S, Jäättelä M (2013). Lysosomal cell death at a glance. J Cell Sci.

[36] Pasquier B (2016). Autophagy inhibitors. Cell Mol Life Sci.

[37] Mauthe M, Orhon I, Rocchi C, Zhou X, Luhr M, Hijlkema KJ, Coppes RP, Engedal N, Mari M, Reggiori F (2018). Chloroquine inhibits autophagic flux by decreasing autophagosome-lysosome fusion. Autophagy.

[38] Zhang Y, Cheng Y, Ren X, Zhang L, Yap KL, Wu H, Patel R, Liu D, Qin ZH, Shih IM, Yang JM (2012). NAC1 modulates sensitivity of ovarian cancer cells to cisplatin by altering the HMGB1-mediated autophagic response. Oncogene.

[39] Liu JT, Li WC, Gao S, Wang F, Li XQ, Yu HQ, Fan LL, Wei W, Wang H, Sun GP (2015). Autophagy inhibition overcomes the antagonistic effect between Gefitinib and Cisplatin in epidermal growth factor receptor mutant non-small-cell lung Cancer cells. Clin Lung Cancer.

[40] Shi S, Tan P, Yan B (2016). ER stress and autophagy are involved in the apoptosis induced by cisplatin in human lung cancer cells. Oncol Rep.

[41] Lin JF, Lin YC, Tsai TF, Chen HE, Chou KY, Hwang IS (2017). Cisplatin induces protective autophagy through activation of BECN1 in human bladder cancer cells. Drug Des Devel Ther.

[42] Schlütermann D, Skowron MA, Berleth N (2018). Targeting urothelial carcinoma cells by combining cisplatin with a specific inhibitor of the autophagy-inducing class III PtdIns3K complex. Urol Oncol Semin Orig Investig.

[43] Fukuda T, Oda K, Wada-Hiraike O, Sone K, Inaba K, Ikeda Y, Miyasaka A, Kashiyama T, Tanikawa M, Arimoto T, Kuramoto H, Yano T, Kawana K, Osuga Y, Fujii T (2015). The anti-malarial chloroquine suppresses proliferation and overcomes cisplatin resistance of endometrial cancer cells via autophagy inhibition. Gynecol Oncol.

[44] Divac Rankov A, Ljujić M, Petrić M, Radojković D, Pešić M, Dinić J (2017). Targeting autophagy to modulate cell survival: a comparative analysis in cancer, normal and embryonic cells. Histochem Cell Biol.

[45] Guo XL, Li D, Hu F, Song JR, Zhang SS, Deng WJ, Sun K, Zhao QD, Xie XQ, Song YJ, Wu MC, Wei LX (2012). Targeting autophagy potentiates chemotherapy-induced apoptosis and proliferation inhibition in hepatocarcinoma cells. Cancer Lett.

[46] Liu M, Bamodu OA, Huang WC, Zucha MA, Lin YK, Wu ATH, Huang CC, Lee WH, Yuan CC, Hsiao M, Deng L, Tzeng YM, Yeh CT (2017). 4-Acetylantroquinonol B suppresses autophagic flux and improves cisplatin sensitivity in highly aggressive epithelial cancer through the PI3K/Akt/mTOR/p70S6K signaling pathway. Toxicol Appl Pharmacol.

[47] Miyamoto M, Takano M, Aoyama T (2018). Phenoxodiol increases cisplatin sensitivity in ovarian clear cancer cells through XIAP down-regulation and autophagy inhibition. Anticancer Res.

[48] Circu M, Cardelli J, Barr M, O’Byrne K, Mills G, el-Osta H (2017). Modulating lysosomal function through lysosome membrane permeabilization or autophagy suppression restores sensitivity to cisplatin in refractory non-small-cell lung cancer cells. PLoS One.

[49] Follo C, Cheng Y, Richards WG, Bueno R, Broaddus VC (2018). Inhibition of autophagy initiation potentiates chemosensitivity in mesothelioma. Mol Carcinog.

[50] Levy JMM, Thorburn A (2012). Modulation of pediatric brain tumor autophagy and chemosensitivity. J Neuro-Oncol.

[51] Yu L, Gu C, Zhong D, Shi L, Kong Y, Zhou Z, Liu S (2014). Induction of autophagy counteracts the anticancer effect of cisplatin in human esophageal cancer cells with acquired drug resistance. Cancer Lett.

[52] Cervia D, Assi E, De Palma C (2016). Essential role for acid sphingomyelinase-inhibited autophagy in melanoma response to cisplatin. Oncotarget.

[53] Wu T, Wang MC, Jing L (2015). Autophagy facilitates lung adenocarcinoma resistance to cisplatin treatment by activation of AMPK/mTOR signaling pathway. Drug Des Devel Ther.

[54] Ojha R, Singh SK, Bhattacharyya S, Dhanda RS, Rakha A, Mandal AK, Jha V (2014). Inhibition of grade dependent autophagy in urothelial carcinoma increases cell death under nutritional limiting condition and potentiates the cytotoxicity of chemotherapeutic agent. J Urol.

[55] Zhang HQ, He B, Fang N, Lu S, Liao YQ, Wan YY (2013). Autophagy inhibition sensitizes cisplatin cytotoxicity in human gastric cancer cell line Sgc7901. Asian Pacific J Cancer Prev.

[56] Ma B, Zhong LL, Qing LG (2013). Inhibition of autophagy enhances cisplatin cytotoxicity in human adenoid cystic carcinoma cells of salivary glands. J Oral Pathol Med.

[57] Bin DZ, Hui B, Shi YH (2011). Autophagy activation in hepatocellular carcinoma contributes to the tolerance of oxaliplatin via reactive oxygen species modulation. Clin Cancer Res.

[58] Klionsky DJ, Elazar Z, Seglen O, Rubinsztein DC (2008). Does bafilomycin a 1 block the fusion of autophagosomes with lysosomes?. Autophagy.

[59] Mauvezin C, Neufeld TP (2015). Bafilomycin A1 disrupts autophagic flux by inhibiting both V-ATPase-dependent acidification and Ca-P60A/SERCA-dependent autophagosome-lysosome fusion. Autophagy.

[60] Chu HY, Wang W, Chen X, Jiang YE, Cheng R, Qi X, Zhong ZM, Zeng MS, Zhu XF, Sun CZ (2018). Bafilomycin A1 increases the sensitivity of tongue squamous cell carcinoma cells to cisplatin by inhibiting the lysosomal uptake of platinum ions but not autophagy. Cancer Lett.

[61] Leisching G, Loos B, Botha M, Engelbrecht AM (2015). A nontoxic concentration of cisplatin induces autophagy in cervical cancer selective cancer cell death with autophagy inhibition as an adjuvant treatment. Int J Gynecol Cancer.

[62] Kang R, Wang ZH, Wang BQ, Zhang CM, Gao W, Feng Y, Bai T, Zhang HL, Huang-Pu H, Wen SX (2012). Inhibition of autophagy-potentiated chemosensitivity to cisplatin in laryngeal cancer Hep-2 cells. Am J Otolaryngol - Head Neck Med Surg.

[63] Hou YJ, Dong LW, Tan YX, Yang GZ, Pan YF, Li Z, Tang L, Wang M, Wang Q, Wang HY (2011). Inhibition of active autophagy induces apoptosis and increases chemosensitivity in cholangiocarcinoma. Lab Investig.

[64] Wang J, Wu GS (2014). Role of autophagy in cisplatin resistance in ovarian cancer cells. J Biol Chem.

[65] Song L, Ma L, Chen G (2016). Autophagy inhibitor 3-methyladenine enhances the sensitivity of nasopharyngeal carcinoma cells to chemotherapy and radiotherapy. Zhong nan Da Xue Xue Bao Yi Xue ban.

[66] Zhang R, Wang R, Chen Q, Chang H (2015). Inhibition of autophagy using 3-methyladenine increases cisplatin-induced apoptosis by increasing endoplasmic reticulum stress in U251 human glioma cells. Mol Med Rep.

[67] Bao L, Jaramillo MC, Zhang Z (2015). Induction of autophagy contributes to cisplatin resistance in human ovarian cancer cells. Mol Med Rep.

[68] Lin WM, Li ZG (2015). Blockage of cisplatin-induced autophagy sensitizes cervical cancer cells to cisplatin. Genet Mol Res.

[69] Jiang L, Huang S, Zhang D, Zhang B, Li K, Li W, Zhang S, Zhang W, Zheng P (2014). Inhibition of autophagy augments chemotherapy in human salivary adenoid cystic carcinoma. J Oral Pathol Med.

[70] Zhang Z, Shao Z, Xiong L, Che B, Deng C, Xu W (2009). Expression of Beclin1 in osteosarcoma and the effects of down-regulation of autophagy on the chemotherapeutic sensitivity. J Huazhong Univ Sci Technol - Med Sci.

[71] Yang Y, Fan Y, Qi Y (2015). Side population cells separated from A549 lung cancer cell line possess cancer stem cell-like properties and inhibition of autophagy potentiates the cytotoxic effect of cisplatin. Oncol Rep.

[72] Sheng J, Shen L, Sun L, et al (2019) Inhibition of PI3K/mTOR increased the sensitivity of hepatocellular carcinoma cells to cisplatin via interference with mitochondrial-lysosomal crosstalk. Cell Prolif 52:(3):e12609. 10.1111/cpr.12609 10.1111/cpr.12609 PMC6536453 31033054

[73] Ko JC, Tsai MS, Chiu YF, Weng SH, Kuo YH, Lin YW (2011). Up-regulation of extracellular signal-regulated kinase 1/2-dependent thymidylate synthase and thymidine phosphorylase contributes to cisplatin resistance in human non-small-cell lung cancer cells. J Pharmacol Exp Ther.

[74] Chen CH, Changou CA, Hsieh TH, Lee YC, Chu CY, Hsu KC, Wang HC, Lin YC, Lo YN, Liu YR, Liou JP, Yen Y (2018). Dual inhibition of PIK3C3 and FGFR as a new therapeutic approach to treat bladder cancer. Clin Cancer Res.

[75] Song H, Pan B, Yi J, Chen L (2014). Featured article: Autophagic activation with Nimotuzumab enhanced chemosensitivity and radiosensitivity of esophageal squamous cell carcinoma. Exp Biol Med.

[76] Ronan B, Flamand O, Vescovi L, Dureuil C, Durand L, Fassy F, Bachelot MF, Lamberton A, Mathieu M, Bertrand T, Marquette JP, el-Ahmad Y, Filoche-Romme B, Schio L, Garcia-Echeverria C, Goulaouic H, Pasquier B (2014). A highly potent and selective Vps34 inhibitor alters vesicle trafficking and autophagy. Nat Chem Biol.

[77] Lyu J, Yang EJ, Head SA, Ai N, Zhang B, Wu C, Li RJ, Liu Y, Yang C, Dang Y, Kwon HJ, Ge W, Liu JO, Shim JS (2017). Pharmacological blockade of cholesterol trafficking by cepharanthine in endothelial cells suppresses angiogenesis and tumor growth. Cancer Lett.

[78] Rena G, Hardie DG, Pearson ER (2017). The mechanisms of action of metformin. Diabetologia.

[79] Saladini S, Aventaggiato M, Barreca F, et al (2019) Metformin impairs glutamine metabolism and autophagy in tumour cells. Cells 14;8(1):49. 10.3390/cells8010049 10.3390/cells8010049 PMC6356289 30646605

[80] Maximchik P, Abdrakhmanov A, Inozemtseva E, Tyurin-Kuzmin PA, Zhivotovsky B, Gogvadze V (2018). 2-Deoxy-D-glucose has distinct and cell line-specific effects on the survival of different cancer cells upon antitumor drug treatment. FEBS J.

[81] Jalota A, Kumar M, Das BC, Yadav AK, Chosdol K, Sinha S (2016). Synergistic increase in efficacy of a combination of 2-deoxy-d-glucose and cisplatin in normoxia and hypoxia: switch from autophagy to apoptosis. Tumor Biol.

[82] Yang Y, Wen FB, Dang LF, Fan Y, Liu D, Wu K, Zhao S (2014). Insulin enhances apoptosis induced by cisplatin in human esophageal squamous cell carcinoma EC9706 cells related to inhibition of autophagy. Chin Med J.

[83] Travelli C, Drago V, Maldi E, Kaludercic N, Galli U, Boldorini R, di Lisa F, Tron GC, Canonico PL, Genazzani AA (2011). Reciprocal potentiation of the antitumoral activities of FK866, an inhibitor of nicotinamide phosphoribosyltransferase, and etoposide or cisplatin in neuroblastoma cells. J Pharmacol Exp Ther.

[84] He H, Jiang H, Chen Y, Ye J, Wang A, Wang C, Liu Q, Liang G, Deng X, Jiang W, Zhou R (2018). Oridonin is a covalent NLRP3 inhibitor with strong anti-inflammasome activity. Nat Commun.

[85] Morré DJ, McClain N, Wu LY, Kelly G, Morré DM (2009). Phenoxodiol treatment alters the subsequent response of ENOX2 (tNOX) and growth of hela cells to paclitaxel and cisplatin. Mol Biotechnol.

[86] Georgaki S, Skopeliti M, Tsiatas M, Nicolaou KA, Ioannou K, Husband A, Bamias A, Dimopoulos MA, Constantinou AI, Tsitsilonis OE (2009). Phenoxodiol, an anticancer isoflavene, induces immunomodulatory effects in vitro and in vivo. J Cell Mol Med.

[87] Zhou J, Hu SE, Tan SH, Cao R, Chen Y, Xia D, Zhu X, Yang XF, Ong CN, Shen HM (2012). Andrographolide sensitizes cisplatin-induced apoptosis via suppression of autophagosome-lysosome fusion in human cancer cells. Autophagy.

[88] Mi S, Xiang G, Yuwen D, Gao J, Guo W, Wu X, Wu X, Sun Y, Su Y, Shen Y, Xu Q (2016). Inhibition of autophagy by andrographolide resensitizes cisplatin-resistant non-small cell lung carcinoma cells via activation of the Akt/mTOR pathway. Toxicol Appl Pharmacol.

[89] Yuwen D, Mi S, Ma Y, Guo W, Xu Q, Shen Y, Shu Y (2017). Andrographolide enhances cisplatin-mediated anticancer effects in lung cancer cells through blockade of autophagy. Anti-Cancer Drugs.

[90] Li J, Yan Y, Sun H, Liu Y, Su CY, Chen HB, Zhang JY (2019). Anti-Cancer effects of Pristimerin and the mechanisms: a critical review. Front Pharmacol.

[91] Zhang Y, Wang J, Hui B, Sun W, Li B, Shi F, Che S, Chai L, Song L (2019). Pristimerin enhances the effect of cisplatin by inhibiting the miR-23a/Akt/GSK3β signaling pathway and suppressing autophagy in lung cancer cells. Int J Mol Med.

[92] Lee Y, Na J, Lee MS, Cha E, Sul J, Park J, Lee J (2018). Combination of pristimerin and paclitaxel additively induces autophagy in human breast cancer cells via ERK1/2 regulation. Mol Med Rep.

[93] Jiang S, Chang H, Deng S, Fan D (2019). Icariin enhances the chemosensitivity of cisplatin-resistant ovarian cancer cells by suppressing autophagy via activation of the AKT/mTOR/ATG5 pathway. Int J Oncol.

[94] Sagrillo-Fagundes L, Bienvenue-Pariseault J, Vaillancourt C (2019). Melatonin: the smart molecule that differentially modulates autophagy in tumor and normal placental cells. PLoS One.

[95] Chen L, Liu L, Li Y, Gao J (2018). Melatonin increases human cervical cancer HeLa cells apoptosis induced by cisplatin via inhibition of JNK/Parkin/mitophagy axis. Vitr Cell Dev Biol - Anim.

[96] Fernandez-Gil BI, Guerra-Librero A, Shen YQ, Florido J, Martínez-Ruiz L, García-López S, Adan C, Rodríguez-Santana C, Acuña-Castroviejo D, Quiñones-Hinojosa A, Fernández-Martínez J, Abdel Moneim AE, López LC, Rodríguez Ferrer JM, Escames G (2019). Melatonin enhances Cisplatin and radiation cytotoxicity in Head and neck squamous cell carcinoma by stimulating mitochondrial ROS generation, apoptosis, and autophagy. Oxidative Med Cell Longev.

[97] Yu F, Liu W, Gong XR (2018). Procyanidins enhance the chemotherapeutic sensitivity of laryngeal carcinoma cells to cisplatin through autophagy pathway. Lin Chung Er bi Yan Hou Tou Jing Wai Ke Za Zhi.

[98] Kadioglu O, Law BYK, Mok SWF, Xu SW, Efferth T, Wong VKW (2017). Mode of action analyses of neferine, a bisbenzylisoquinoline alkaloid of lotus (Nelumbo nucifera) against multidrug-resistant tumor cells. Front Pharmacol.

[99] Kalai Selvi S, Vinoth A, Varadharajan T, Weng CF, Vijaya Padma V (2017). Neferine augments therapeutic efficacy of cisplatin through ROS- mediated non-canonical autophagy in human lung adenocarcinoma (A549 cells). Food Chem Toxicol.

[100] Zhu X, Ji M, Han Y, Guo Y, Zhu W, Gao F, Yang X, Zhang C (2017). PGRMC1-dependent autophagy by hyperoside induces apoptosis and sensitizes ovarian cancer cells to cisplatin treatment. Int J Oncol.

[101] Yu N, Xiong Y, Wang C (2017). Bu-Zhong-Yi-Qi decoction, the water extract of Chinese traditional herbal medicine, enhances Cisplatin cytotoxicity in A549/DDP cells through induction of apoptosis and autophagy. Biomed Res Int.

[102] Dyshlovoy SA, Hauschild J, Amann K (2015). Marine alkaloid Monanchocidin a overcomes drug resistance by induction of autophagy and lysosomal membrane permeabilization. Oncotarget.

[103] Hu F, Wei F, Wang Y, Wu B, Fang Y, Xiong B (2015). EGCG synergizes the therapeutic effect of cisplatin and oxaliplatin through autophagic pathway in human colorectal cancer cells. J Pharmacol Sci.

[104] Shi S, Wang Q, Xu J (2015). Synergistic anticancer effect of cisplatin and Chal-24 combination through IAP and c-FLIPL degradation, Ripoptosome formation and autophagy-mediated apoptosis. Oncotarget.

[105] Varoni EM, Lo Faro AF, Sharifi-Rad J, Iriti M (2016). Anticancer molecular mechanisms of resveratrol. Front Nutr.

[106] Fei Q, Kent D, Botello-Smith WM, Nur F, Nur S, Alsamarah A, Chatterjee P, Lambros M, Luo Y (2018). Molecular mechanism of Resveratrol’s lipid membrane protection. Sci Rep.

[107] Hu S, Li X, Xu R (2016). The synergistic effect of resveratrol in combination with Cisplatin on apoptosis via modulating autophagy in A549 cells. Acta Biochim Biophys Sin Shanghai.

[108] Zhao T, Wang HJ, Zhao WW, Sun YL, Hu LK (2017). Gambogic acid improves non-small cell lung cancer progression by inhibition of mTOR signaling pathway. Kaohsiung J Med Sci.

[109] Hsin IL, Ou CC, Wu MF, Jan MS, Hsiao YM, Lin CH, Ko JL (2015). GMI, an immunomodulatory protein from ganoderma microsporum, potentiates cisplatin-induced apoptosis via autophagy in lung cancer cells. Mol Pharm.

[110] Ur Rahman MS, Zhang L, Wu L, Xie Y, Li C, Cao J (2017). Sensitization of gastric cancer cells to alkylating agents by glaucocalyxin B via cell cycle arrest and enhanced cell death. Drug Des Devel Ther.

[111] Zajdel A, Wilczok A, Latocha M, Tarkowski M, Kokocińska M, Dzierzewicz Z (2014). Polyunsaturated fatty acids potentiate cytotoxicity of Cisplatin in A549 cells. Acta Pol Pharm.

[112] Potočnjak I, Šimić L, Vukelić I, Domitrović R (2019). Oleanolic acid attenuates cisplatin-induced nephrotoxicity in mice and chemosensitizes human cervical cancer cells to cisplatin cytotoxicity. Food Chem Toxicol.

[113] Khurana A, Roy D, Kalogera E (2015). Quinacrine promotes autophagic cell death and chemosensitivity in ovarian cancer and attenuates tumor growth. Oncotarget.

[114] Bryant J, Batis N, Franke AC (2019). Repurposed quinacrine synergizes with cisplatin, reducing the effective dose required for treatment of head and neck squamous cell carcinoma. Oncotarget.

[115] Piperno A, Scala A, Mazzaglia A, Neri G, Pennisi R, Sciortino M, Grassi G (2018). Cellular signaling pathways activated by functional graphene nanomaterials. Int J Mol Sci.

[116] Lin KC, Lin MW, Hsu MN, Yu-Chen G, Chao YC, Tuan HY, Chiang CS, Hu YC (2018). Graphene oxide sensitizes cancer cells to chemotherapeutics by inducing early autophagy events, promoting nuclear trafficking and necrosis. Theranostics.

[117] Kao C, Chao A, Tsai CL, Chuang WC, Huang WP, Chen GC, Lin CY, Wang TH, Wang HS, Lai CH (2014). Bortezomib enhances cancer cell death by blocking the autophagic flux through stimulating ERK phosphorylation. Cell Death Dis.

[118] Ge XY, Yang LQ, Jiang Y, Yang WW, Fu J, Li SL (2014). Reactive oxygen species and autophagy associated apoptosis and limitation of clonogenic survival induced by zoledronic acid in salivary adenoid cystic carcinoma cell line SACC-83. PLoS One.

[119] O’Donovan TR, Rajendran S, O’Reilly S, O’Sullivan GC, McKenna SL (2015). Lithium modulates autophagy in esophageal and colorectal cancer cells and enhances the efficacy of therapeutic agents in vitro and in vivo. PLoS One.

[120] Wei P, Zhang L, Lu Y, Man N, Wen L (2010). C60(Nd) nanoparticles enhance chemotherapeutic susceptibility of Cancer cells by modulation of autophagy. Nanotechnology.

[121] Hou H, Zhang Y, Huang Y, Yi Q, Lv L, Zhang T, Chen D, Hao Q, Shi Q (2012). Inhibitors of phosphatidylinositol 3′-kinases promote mitotic cell death in HeLa cells. PLoS One.

[122] Pike KG, Malagu K, Hummersone MG, Menear KA, Duggan HME, Gomez S, Martin NMB, Ruston L, Pass SL, Pass M (2013). Optimization of potent and selective dual mTORC1 and mTORC2 inhibitors: the discovery of AZD8055 and AZD2014. Bioorganic Med Chem Lett.

[123] Mallon R, Hollander I, Feldberg L, Lucas J, Soloveva V, Venkatesan A, Dehnhardt C, Delos Santos E, Chen Z, dos Santos O, Ayral-Kaloustian S, Gibbons J (2010). Antitumor efficacy profile of PKI-402, a dual phosphatidylinositol 3-kinase/mammalian target of rapamycin inhibitor. Mol Cancer Ther.

[124] Morrison DK (2012). MAP kinase pathways. Cold Spring Harb Perspect Biol.

[125] Sarkar S, Carroll B, Buganim Y, Maetzel D, Ng AHM, Cassady JP, Cohen MA, Chakraborty S, Wang H, Spooner E, Ploegh H, Gsponer J, Korolchuk VI, Jaenisch R (2013). Impaired autophagy in the lipid-storage disorder niemann-pick type c1 disease. Cell Rep.

[126] Yao Z, Xie F, Li M, Liang Z, Xu W, Yang J, Liu C, Li H, Zhou H, Qu LH (2017). Oridonin induces autophagy via inhibition of glucose metabolism in p53-mutated colorectal cancer cells. Cell Death Dis.

[127] Zhao Y, Xia H (2019). Oridonin elevates sensitivity of ovarian carcinoma cells to cisplatin via suppressing cisplatin-mediated autophagy. Life Sci.

[128] Yang H, Gao Y, Fan X, Liu X, Peng L, Ci X (2019). Oridonin sensitizes cisplatin-induced apoptosis via AMPK/Akt/mTOR-dependent autophagosome accumulation in A549 cells. Front Oncol.

[129] Kochetkova EY, Blinova GI, Zubova SG, Bykova TV, Pospelov VA, Pospelova TV (2017). The MEK/ERK pathway is essential for maintenance of cytoprotective autophagy in E1A+cHA-RAS transformants after exposure to radiation. Cell Tissue Biol.

[130] Wang J, Whiteman MW, Lian H, Wang G, Singh A, Huang D, Denmark T (2009). A non-canonical MEK/ERK signaling pathway regulates autophagy via regulating Beclin 1. J Biol Chem.

[131] Bordoni A, Di Nunzio M, Danesi F, Biagi PL (2006). Polyunsaturated fatty acids: from diet to binding to ppars and other nuclear receptors. Genes Nutr.

[132] Buckingham EM, Carpenter JE, Jackson W, Grose C (2014). Nuclear LC3-positive puncta in stressed cells do not represent autophagosomes. Biotechniques.

[133] Morozov AV, Karpov VL (2019). Proteasomes and several aspects of their heterogeneity relevant to cancer. Front Oncol.

[134] Russ KA, Elvati P, Parsonage TL, Dews A, Jarvis JA, Ray M, Schneider B, Smith PJS, Williamson PTF, Violi A, Philbert MA (2016). C60 fullerene localization and membrane interactions in RAW 264.7 immortalized mouse macrophages. Nanoscale.

[135] Duan G, Song Z, Qi M, Bai X, Wang J, Zhang Y, Zou X, Guo Q, Wan P (2018). Increased autophagy levels mediate Cisplatin resistance in Cisplatin-resistant cells while also rendering them vulnerable to autophagy induction. Biomed Res Int.

[136] Kucharewicz K, Dudkowska M, Zawadzka A, Ogrodnik M, Szczepankiewicz AA, Czarnocki Z, Sikora E (2018). Simultaneous induction and blockade of autophagy by a single agent. Cell Death Dis.

